# Conserved-Potential-Driven Molecular Dynamics Deciphers Formose Reaction Mechanisms

**DOI:** 10.1021/jacsau.5c01359

**Published:** 2026-01-22

**Authors:** Hei Wun Kan, Xiao-Tian Li, Tong Zhu, Yuzhi Xu, John Zeng Hui Zhang

**Affiliations:** † Faculty of Synthetic Biology, 85411 Shenzhen University of Advanced Technology, Shenzhen 518107, China; ‡ Shanghai Engineering Research Center of Molecular Therapeutics and New Drug Development, School of Chemistry and Molecular Engineering, 12655 East China Normal University, Shanghai 200062, China; § NYU-ECNU Center for Computational Chemistry, NYU Shanghai, Shanghai 200126, China; ∥ Department of Chemistry, 5894 New York University, New York, New York 10003, United States; ⊥ Collaborative Innovation Center of Extreme Optics, Shanxi University, Taiyuan 030006, China

**Keywords:** RTIP-MD, formose reaction, formaldehyde self-condensation, autocatalytic cycle, ribose synthesis

## Abstract

The formose reaction, in which formaldehyde reacts to form sugars under alkaline conditions, is a leading candidate for prebiotic sugar synthesis, with ribose as a particularly significant though minor product. Despite the simplicity of its starting material (formaldehyde), the reaction involves intricate mechanistic steps and generates a complex product mixture, hindering full mechanistic elucidation even after decades of study. Here, we develop an efficient, mechanism-free molecular dynamics (MD) approach to simulate the formose reaction, using our recently proposed roto-translationally invariant potential (RTIP) to drive the molecular system toward reactive configurations for potential reactions. High-resolution RTIP-MD trajectories reveal a comprehensive reaction network, elucidating previously elusive mechanistic details for formaldehyde self-condensation, aldose-ketose tautomerization, and ribose synthesis. Based on the Gibbs free energy landscape, the microkinetic simulation conclusively settles the autocatalytic cycle debate, demonstrating that autocatalysis occurs predominantly at low glycolaldehyde concentrations, as evidenced by the reverse aldotetrose retroaldol cleavage. This proof-of-concept study demonstrates RTIP-MD’s capability to simulate complex, multistep reactions, suggesting potential applicability to challenging systems such as enzyme catalysis.

## Graphical abstract

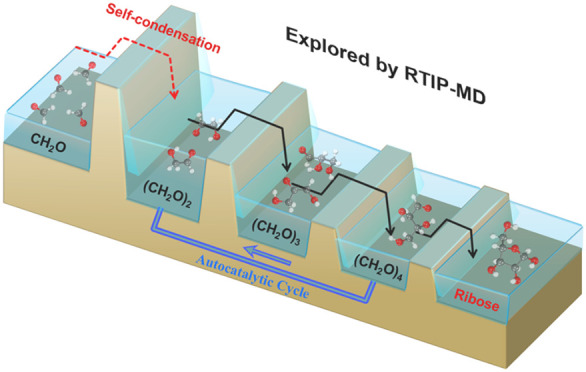

## 1 Introduction

Because of RNA’s diverse biological functions, the “RNA world” hypothesis^1,2^ remains the leading model for life’s chemical origins, despite persistent challenges in prebiotic synthesis, particularly for ribose, a key RNA component. While the well-known formose reaction provides a plausible prebiotic route to various sugars, including ribose, its intricate reaction network, kinetics, and products remain elusive despite extensive research.^3–5^ This reaction, first reported by Butlerov in 1861,^6^ involves the base-catalyzed condensation of formaldehyde (CH_2_O) in aqueous solution, yielding a complex mixture of sugar-like compounds, (CH_2_O)_*n*_, including glycolaldehyde (HOCH_2_CHO) and other carbohydrates. Since formaldehyde, the feedstock of the formose reaction, is known to be an important product in many prebiotic experiments and theoretical studies,^7–13^ the key challenge lies in establishing the rationale for prebiotic spontaneous synthesis. To this end, various minerals, such as borate,^14,15^ silicate,^16,17^ and metal-doped-clays,^18^ have been proposed to stabilize pentoses (particularly ribose) by forming complexes, addressing their typically low yields (<1%) and poor stability in the formose reaction.^5^ Additionally, Gardner et al. demonstrated that carbohydrate synthesis from formaldehyde could also proceed within a lipid vesicle, representing a step toward the design of artificial cells from simple chemical building blocks.^19^ More importantly, the significant analytical challenge posed by the complex product mixture of the formose reaction has driven numerous excellent experimental studies in recent years: for example, Huck et al.^20–22^ employed a combination of gas/liquid chromatography–mass spectrometry to investigate the self-organization in the formose reaction under varied environmental conditions; Briš et al.^23^ designed ion mobility separation to replace chromatographic separation to enable online monitoring; more subtly, Krishnamurthy et al.^24^ employed ^13^C nuclear magnetic resonance to perform online analysis of C–C bonding, revealing the complex condensation mechanism and selectivity under mildly alkaline, bicarbonate-buffered conditions (pH 8.5). Despite these advances, the primitive formose reaction, however, remains poorly understood and continues to pose major challenges, as introduced below.

In the elementary formose reaction, formaldehyde, as the sole feedstock, is added to an alkaline aqueous solution (pH 10–12) and catalyzed by metal ions (typically Ca^2+^) at mild temperatures (40–70 °C); the initial dimerization of formaldehyde to glycolaldehyde proceeds very slowly, leading to a long induction period; once glycolaldehyde is formed, it rapidly reacts with more formaldehyde, producing a wide variety of carbohydrates; as the reaction progresses, the formaldehyde concentration decreases dramatically and the solution turns yellow, indicating the formation of structurally unresolved polymeric products.^6,15,20,21,25–28^ Underlying the complex processes, several problems have long puzzled researchers, regarding the reaction mechanism and kinetics. First, as an electrophilic species, formaldehyde lacks a natural tendency to undergo self-condensation. How the initial dimerization occurs remains unclear, which may involve umpolung of one formaldehyde molecule.^5,25,27,28^ Second, to interpret the sharp consumption of formaldehyde after the induction period, Breslow proposed a well-known autocatalytic cycle mechanism in 1959, which involves three types of reactions: (i) nucleophilic attack of deprotonated glycolaldehyde on a carbonyl electrophile to form a new C–C bond (aldol reaction); (ii) tautomerization between aldoses and ketoses via enolization (e.g., interconversion of glyceraldehyde and dihydroxyacetone, ketotetrose and aldotetrose); (iii) retroaldol cleavage of aldotetrose to yield two molecules of glycolaldehyde, which can initiate the next cycle.^25^ This established an alternative glycolaldehyde synthesis pathway that bypassed the kinetically limited dimerization of formaldehyde, thereby providing a qualitative explanation for the observed cascading acceleration of reaction rates after the induction period. Beyond this simplest cycle, Benner’s group^15^ identified numerous retroaldolization reactions from branched carbohydrates, posing a challenge to validate the autocatalytic nature. Third, Cannizzaro-type disproportionation may compete with the formose condensation, yielding unreactive formic acid and methanol, further complicating the reaction network.^3,5,15,20^

Alongside experimental studies, density functional theory (DFT) calculations have been widely employed to investigate the formose reaction mechanism and its energetic landscape.^29–38^ For example, early in 2013, Kua et al. had calculated formaldehyde oligomerization in neutral aqueous solution, using water molecules as catalytic proton shuttles.^31^ The results showed that the vital formaldehyde dimerization to glycolaldehyde has an extremely high barrier of 45.3 kcal mol^–1^ without the catalysis of metal ions and alkali.^31^ Later, Thripati et al. presented a synergistic metal-ion and hydrogen-bond-mediated mechanism for the gas-phase conversion of formaldehyde to glycolaldehyde, but this pathway appears specific to interstellar prebiotic chemistry.^32^ More significantly, Jeilani et al. proposed a free radical pathway for the synthesis of ribose and RNA nucleoside catalyzed by Ca^2+^ and CaOH^+^, using ^•^CH_2_OH as a feedstock.^35^ Although this pathway exhibits a reasonable barrier of 25.1 kcal mol^–1^ for the initial formaldehyde dimerization, it cannot adequately explain the natural generation of ^•^CH_2_OH, which requires overcoming a high barrier of 41.0 kcal mol^–1^.^35^ Regarding autocatalytic cycle, to the best of our knowledge, only Tabata et al. have recently reported a routine free energy comparison between basic (NaOH-catalyzed) and neutral (Na_2_WO_4_-catalyzed) conditions.^37^ In contrast to comprehensive experimental studies, conventional theoretical approaches remain inadequate, constrained in their capacity to elucidate the fundamental mechanisms of the intricate formose reaction.

In this study, we develop an efficient molecular dynamics (MD) method driven by our recently proposed roto-translationally invariant potential (RTIP), enabling automated, mechanism-free simulations of complex reaction dynamics.^13^ By analyzing the RTIP-MD trajectories, we construct a comprehensive reaction network for the formose reaction, involving formaldehyde self-condensation, aldose-ketose tautomerization, and ribose synthesis. Through transition state (TS) searches, DFT calculations, and thermodynamic corrections, we establish the Gibbs free energy profiles for the reaction network, allowing a detailed microkinetic simulation. The simulation results reveal previously inaccessible kinetic details, providing mechanistic explanations for several puzzling experimental observations. Alongside the crucial formose reaction, we have extended our research by applying the RTIP-MD method to investigate the Cannizzaro-type side reactions, which yield byproducts such as formic acid, methanol, carbon monoxide, carbon dioxide, and hydrogen gas.^39^ This companion study is essential for a complete understanding of the whole reaction network.

## 2 Methods

### 2.1 Computational Model

From RTIP-MD trajectories, we identified frequent neutral-monoanion interconversions near Ca^2+^ ions, mediated by proton transfer (e.g., H_2_O ⇌ OH^–^ + H^+^ and CH_2_O ⇌ CHO^–^ + H^+^). These neutral-monoanion interconversions are a key characteristic of the formose reaction, as they are more efficient than the H_2_O-mediated mechanisms under neutral conditions. To incorporate the catalytic role of Ca^2+^ and OH^–^, and maintain electroneutrality, we constructed a uniform computational model for DFT calculations. This model contains exactly one Ca^2+^ and one OH^–^ as catalytic species, along with one monoanion and an additional molecule (when present) as reactants or products. For instance, the reaction network in Figure 1a begins with a Ca^2+^-OH^–^-OH^–^-CH_2_O complex, with Ca^2+^-OH^–^ pair as the catalytic species and OH^–^-CH_2_O pair as the reactants.

**Figure 1:**
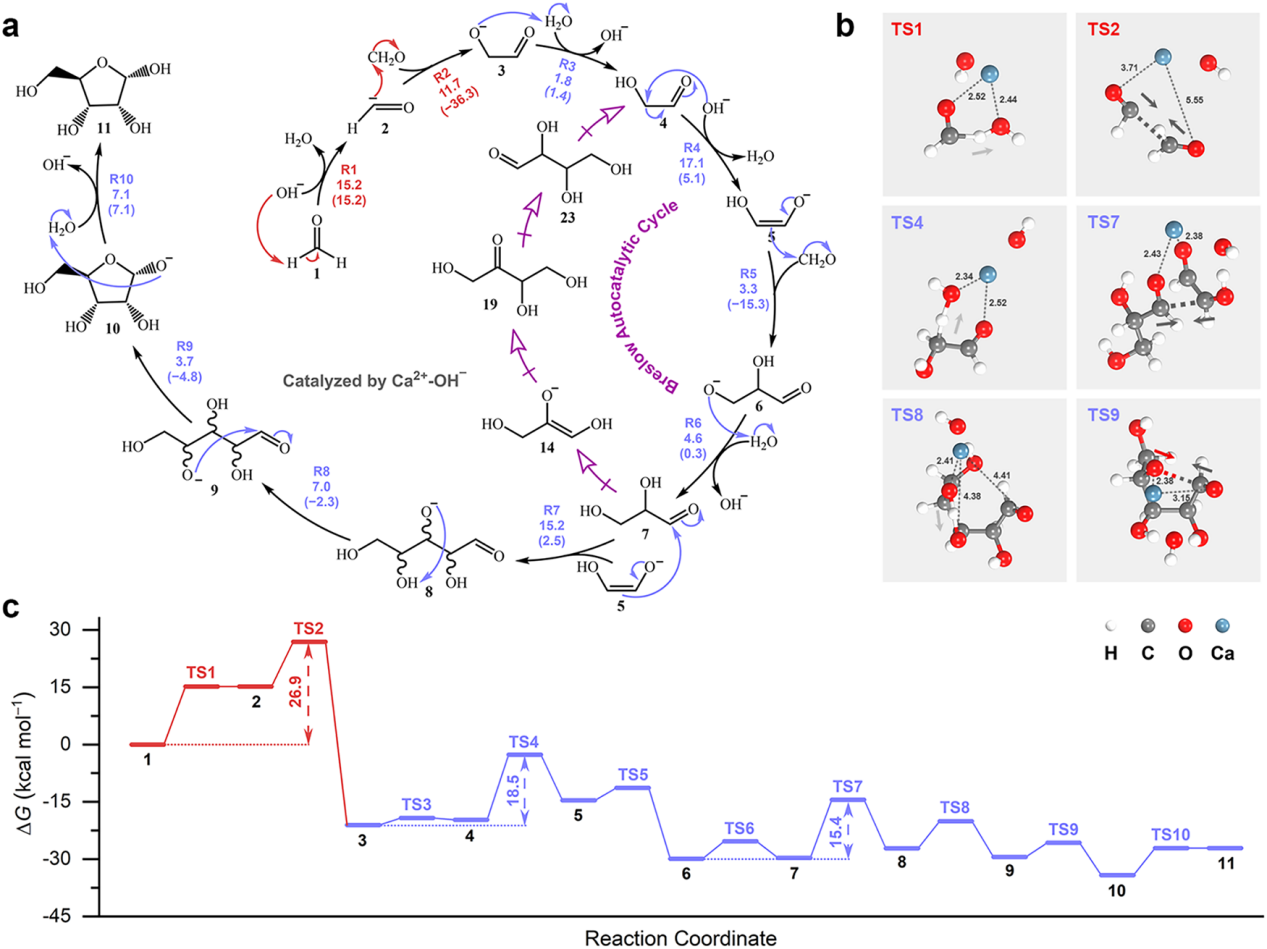
Mechanistic details of ribose synthesis in the formose reaction. (a) Formose reaction network tracing ribose synthesis. For clarity, each species is labeled with a bold number (e.g., **1** for formaldehyde, **2** for formyl anion), and each reaction step is assigned an index (e.g., R1 for deprotonation of **1**, R2 for coupling of **1** and **2**). The activation free energy barrier (*G*_a_, kcal mol^–1^) and reaction free energy (Δ*G*, kcal mol^–1^; in parenthesis) are provided for each reaction step, representing their kinetic and thermodynamic properties. Although depicted with unidirectional arrows, all reactions are kinetically reversible, as catalyzed by the Ca^2+^-OH^–^ complex (see Section 2 for details). (b) TS structures for the key steps selected from the reaction network. The arrows depict the imaginary-frequency vibrational mode at TS, illustrating the atomic displacements along the reaction coordinate. The Ca-O distances for the carbonyl and OH^–^ groups are indicated in Å. (c) Gibbs free energy profiles (at 55 °C, 1 atm, 1 mol L^–1^) for ribose synthesis, with rate-determining steps highlighted in red.

### 2.2 TS Search

Using the established computational model, we carried out high-accuracy DFT calculations to determine the structures and energetics of mechanistically important reaction steps identified in RTIP-MD trajectories. For each step, the initial state, final state, and TS were determined as follows: (i) first, the nudged elastic band (NEB) method^40,41^ was utilized to systematically optimize the reaction pathway; (ii) next, the TS structure was precisely located using the dimer method,^42^ with verification of its imaginary-frequency vibrational mode; (iii) subsequently, the reactant and product structures were obtained by extrapolation optimizations along the imaginary-frequency vibrational direction; (iv) finally, thermodynamic corrections (at 55 °C, 1 atm, 1 mol L^–1^) were applied to the reactant, product, and TS structures based on vibrational frequency calculations performed with the Shermo program.^43^

### 2.3 DFT Calculations

The DFT calculations were performed using the Gaussian and plane waves (GPW) method, as implemented in the Quickstep electronic structure module^44^ of CP2K (version 2022.1).^45^ The TZVP Gaussian basis set^46^ was used for molecular orbitals, while a 1 × 1 × 1 *k*-point mesh was employed for plane waves. Electronic exchange and correlation were treated with the ωB97M-V functionals,^47^ combined with VV10 nonlocal correlation.^48^ Solvation effects were incorporated using the self-consistent continuum solvation (SCCS) model,^49^ with water as the solvent. The optimization convergence criteria were: maximum force ≤ 0.0002 hartree/bohr, root-mean-square (RMS) force ≤ 0.0001 hartree/bohr, maximum displacement ≤ 0.002 bohr, and RMS displacement ≤ 0.001 bohr.

### 2.4 Microkinetic Simulation

A straightforward microkinetic model, grounded in transition state theory, was designed to simulate the time evolution of the formose reaction. The program is available on GitHub (https://github.com/MillenniumDream/Microkinetics).

In the formose reaction network, many proton transfer steps (see Figures 1 and 2) are exceptionally fast, with characteristic timescales orders of magnitude shorter than those of the rate-determining steps. This disparity creates a stringent bottleneck on the simulation time step. To enable an efficient time step of 5.0 × 10^–12^ s, the free energy barriers for these rapid proton transfers were uniformly elevated to ≥ 5.0 kcal mol^–1^ in both forward and reverse directions (see Table S1). This artificial elevation locally lengthens the timescales of these local processes while preserving the overall reaction thermodynamics (exothermicity/endothermicity) and kinetics (timescales for those pivotal conversions). Conceptually analogous to the SHAKE constraint algorithm in MD, both circumventing fast but trivial modes, this adjustment allowed the microkinetic simulation to be completed within days.

**Figure 2:**
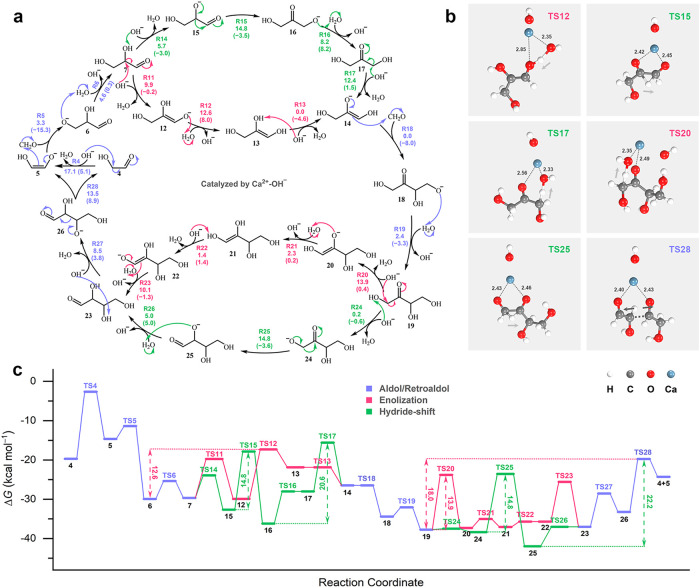
Mechanistic details of Breslow autocatalytic cycle in the formose reaction. (a) Stepwise reaction network of the Breslow autocatalytic cycle, with the aldol/retroaldol reaction (blue), enolization-induced isomerization (pink), and hydride-shift isomerization (green) highlighted in different colors. The other symbols/labels remain consistent as in Figure 1. (b) TS structures for the key steps selected from the reaction network. (c) Gibbs free energy profiles (at 55 °C, 1 atm, 1 mol L^–1^) for the Breslow autocatalytic cycle.

## 3 Results and Discussion

### 3.1 RTIP-MD Simulations

To resolve the intricate formose reaction mechanism, we integrated our newly developed RTIP method^13^ with conventional MD, enabling efficient and thorough exploration of the vast reaction space. The RTIP, by definition, is formulated using a metric derived from pristine Cartesian coordinates, inherently preserving roto-translational invariance (see Supporting Methods).^13^ Compared with traditional internal coordinates (e.g., bond lengths/angles), RTIP shows superior tractability owing to its Cartesian-coordinate basis. In this study, we designed a Gaussian-type oscillating RTIP to automatically steer molecular species toward reactive configurations in MD simulations, facilitating potential reactions (see Figure 3), as detailed below.

**Figure 3:**
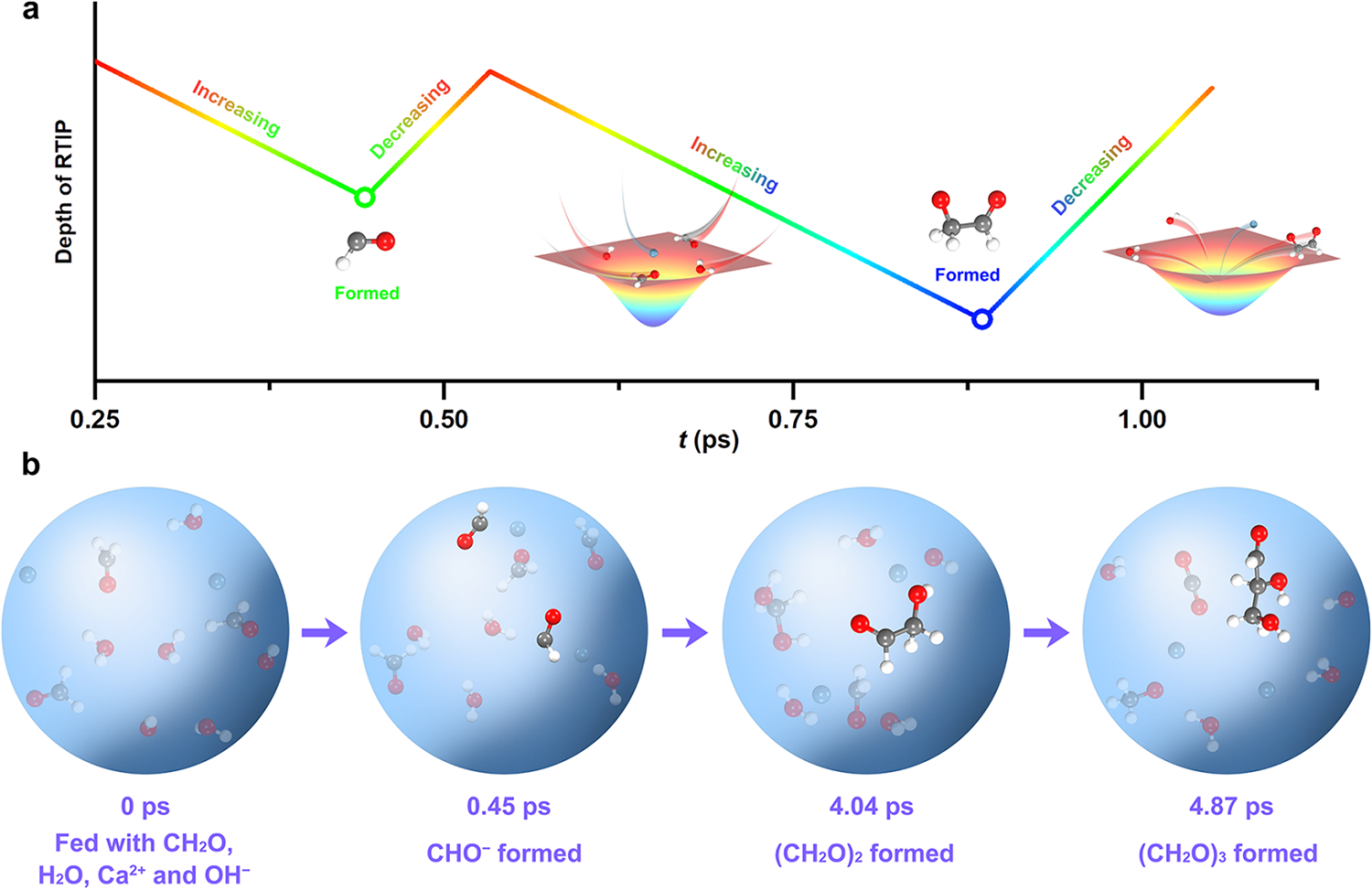
Schematic diagram of RTIP-MD approach. (a) Progression of a typical RTIP-MD simulation demonstrating the successive synthesis of formyl anion (**2**) and glycolaldehyde alkoxide anion (**3**). The well depth of the Gaussian-type attractive RTIP is periodically modulated, facilitating both product synthesis and dispersal. (b) Snapshots of a typical RTIP-MD simulation illustrating the successive synthesis of formyl anion (**2**), glycolaldehyde (**4**), and glyceraldehyde (**7**; Supporting Video 1).

At the start of the reaction search, the reactants are positioned at a separation distance, with their initial bonding configurations recorded as reference. Next, a Gaussian-type attractive RTIP is applied to the reactants, with its depth increasing linearly over time to gradually drive the molecular species toward reactive proximity (see the schematic diagram in Figure 3a). Upon detection of atomic bonding changes, indicating product formation (e.g., the formyl anion and glycolaldehyde alkoxide anion synthesized in Figure 3a), the RTIP immediately turns decreasing at twice speed to separate the species. When the RTIP depth reaches the lower threshold, the atomic bonding configurations are updated, triggering a renewed RTIP increase for the next reaction search cycle.

Additionally, a tailored bond-monitoring scheme was implemented to track the desired condensation and autocatalysis in the formose reaction. Specifically, the current system comprises four elements (C, H, O, and Ca), including ten distinct bond types: C–C, C–H, C–O, C–Ca, H–H, H–O, H–Ca, O–O, O–Ca, and Ca–Ca. Among these bonds, changes in C–Ca, H–Ca, O–Ca, and Ca–Ca reflect complexation states; changes in C–O bonds signal the formation/dissociation of aldehyde hydrate and ether; while changes in H–O bonds correlate with the protonation/deprotonation equilibrium of hydroxyl groups (e.g., OH^–^ + H^+^ ⇌ H_2_O). Because of the insignificance of these reactions, their characteristic bond changes (C–Ca, H–Ca, O–Ca, Ca–Ca, C–O, and H–O) were not monitored in this study. Only changes in the remaining bonds (C–C, C–H, H–H, and O–O) were detected for RTIP control, with their corresponding reactions identified as: C–C bonds for aldol/retroaldol reactions; C–H bonds for enolization (deprotonation at the C atom next to carbonyl group); H–H bonds for H_2_ formation; and O–O bonds for no detectable reaction. Overall, the bond-monitoring scheme can bias the MD simulations to favor the formose reaction (signaled by changes in C–C and C–H bonds) over the Cannizzaro-type disproportionation (signaled by changes in C–O bonds).

In practice, the oscillating Gaussian-type RTIP was implemented with the computationally efficient B97–3c density functional and the D3 semiclassical dispersion correction. The canonical ensemble MD (NVT) was performed using a Berendsen thermostat maintained at a high temperature of 1500 K to ensure comprehensive sampling of the reaction space. This specific temperature was selected based on rigorous testing, which revealed the following progression: at 500 K, only trivial proton exchange and H_2_COOH^–^ formation occur on a picosecond time scale; at 1000 K, the Cannizzaro reaction pathway activates; and accessing the formose reaction requires the target temperature of 1500 K. Figure 3b schematically displays the evolution of a typical simulation cell containing 8 H_2_O, 8 CH_2_O, 2 Ca^2+^, and 4 OH^–^, showing the successive synthesis of formyl anion, glycolaldehyde, and glyceraldehyde over 5 ps (10000 steps at 0.5 fs/step; see Supporting Video 1). To broaden the reaction search and suppress the competing Cannizzaro reaction, some key intermediates (e.g., glycolaldehyde, glyceraldehyde, and aldotetrose) were introduced as initial components in follow-up simulations (see Supporting Videos 2, 3, 4, 5, 6, 7).

The RTIP steering and high temperature of 1500 K, on the one hand effectively improve the reaction sampling, but on the other hand induce some issues, as briefly discussed here. First, the extreme simulation conditions and the simplified models (e.g., containing only 8 H_2_O, 8 CH_2_O, 2 Ca^2+^, and 4 OH^–^) differ significantly from those in typical formose experiments, a discrepancy that can influence reaction selectivity and kinetics to some extent. To better capture realistic reaction behavior, we therefore employ the ωB97M-V density functional to calculate Gibbs free energy profiles for the key reaction pathways (see Section 2), and subsequently perform a microkinetic simulation to model the reaction evolution under experimental conditions (see below). Second, severe configurational distortion under the extreme conditions makes it difficult to distinguish diastereomers; therefore, they are not treated separately in this study.

### 3.2 Synthesis of Ribose

Figure 1 presents a global reaction network for the formose reaction, with the synthesis of ribose illustrated in detail. As noted, a key step turns out to be the initial dimerization of formaldehyde to glycolaldehyde, which involves umpolung of the electrophilic carbon in one of formaldehyde molecules. From the RTIP-MD trajectories (Supporting Video 1), we observed the formaldehyde molecule (**1**) losing a proton near Ca^2+^, concurrent with the conversion of OH^–^ to H_2_O, despite the significant endothermicity in Gibbs free energy (15.2 kcal mol^–1^; see R1 in Figure 1a and the corresponding TS1 in Figure 1b). The generated formyl anion (**2**) is nucleophilic and thus may overcome an additional barrier of 11.7 kcal mol^–1^ to attack formaldehyde, forming glycolaldehyde alkoxide anion (**3**, R2). The coupling, however, proceeds through an unstable TS with weak Ca–O coordination (see Figure 1b), resulting in a high overall barrier of 26.9 kcal mol^–1^. The two steps, as highlighted in red in Figure 1, turn out to be rate-determining throughout the formose reaction. Subsequently, intermediate **3** can easily abstract a proton from H_2_O to yield glycolaldehyde (**4**) while regenerating OH^–^ (R3). In total, the formaldehyde dimerization is strongly exothermic by 19.7 kcal mol^–1^ (Figure 1c), primarily due to the C–C bond formation.

Once formed, glycolaldehyde may readily undergo aldol additions with more formaldehyde molecules, producing trioses, tetroses, and pentoses, as introduced below. Under alkaline conditions, glycolaldehyde (**4**) is likely to transfer a proton to OH^–^ at the C atom next to the carbonyl group (enolization), forming a resonance-stabilized carbanion/oxyanion, with a moderate free energy barrier of 18.5 kcal mol^–1^ (R4). The formed glycolaldehyde enolate anion (**5**) then can nucleophilically attack formaldehyde, generating the glyceraldehyde alkoxide anion (**6**, R5), the precursor to glyceraldehyde (**7**). The addition reaction is thermodynamically favorable, with an exothermicity of 10.0 kcal mol^–1^ (Figure 1c).

In the subsequent conversion of triose (glyceraldehyde) to pentose, the RTIP-MD trajectories (Supporting Video 2) reveal a characteristic C3 → C2 → C5 pathway, consistent with recent triose-initiated formose experiments:^23^ glyceraldehyde (**7**) first undergoes retroaldol cleavage to yield the glycolaldehyde enolate anion (**5**), which then nucleophilically attacks a second glyceraldehyde (**7**) to generate the linear pentose alkoxide anion (**8**, R7; *G*_a_ = 15.4 kcal mol^–1^), thereby bypassing tetrose formation. Next, via proton transfer, intermediate **8** can readily convert to another pentose alkoxide anion (**9**, R8), and further cyclize to yield the furanose alkoxide anion **10** (R9; Supporting Video 3). The protonation of **10** eventually achieves ribose (**11**, R10). Compared to glyceraldehyde, ribose is slightly more stable by 4.6 kcal mol^–1^ in free energy (Figure 1c).

As aldol additions are flexible, ribose could also arise from a gradual buildup, where a C4 ketose undergoes an aldol reaction with formaldehyde to give a 3-ketopentose, followed by aldose–ketose isomerization via enediol chemistry. However, recent isotope-labeling experiments^23^ reveal that this C4 + C1 route is thermodynamically less favorable than the direct C3 + C2 aldol alternative for the synthesis of pentoses.

### 3.3 Breslow Autocatalytic Cycle

To explain the cascade of aldol reactions after the induction period, Breslow proposed a well-known autocatalytic cycle mechanism in 1959 to account for the rapid generation of glycolaldehyde rather than the unfavorable formaldehyde dimerization.^25,28,50^ Indeed, our RTIP-MD simulations have captured both aldose-ketose tautomerization (via enolization^25^ or hydride-shift^28,50^ ) and retroaldol cleavage of aldotetrose (Supporting Videos 4, 5, 6, 7), as summarized in Figures 2, S2, and S3.

In the initial stage of the Breslow autocatalytic cycle, glycolaldehyde (**4**) undergoes successive enolization (R4), formaldehyde addition (R5), and protonation (R6) to yield glyceraldehyde (**7**), as introduced above (Figure 2a). Next, glyceraldehyde (**7**) needs to convert to the dihydroxyacetone enolate anion (**14**) via two possible mechanistic pathways. In the enolization pathway (pink), glyceraldehyde (**7**) undergoes C-2 deprotonation (R11) followed by C-1 oxyanion protonation (R12) to form enol dehydroglycerol (**13**), which is significantly less stable than its aldose and ketose tautomers (**7** and **17**; see Figure 2c). With an endothermicity of 8.0 kcal mol^–1^ (R12), the process exhibits an overall free energy barrier of 12.6 kcal mol^–1^ (Figure 2c). Subsequently, enol dehydroglycerol (**13**) can easily convert to dihydroxyacetone enolate anion (**14**) via deprotonation of the C-2 hydroxyl group. Alternatively, in the hydride-shift pathway (green), the protonation/deprotonation at the hydroxyl group (R14, 16) turns out to be trivial. The characteristic step involves a direct 1,2-hydride shift (from C-2 to C-1) with a moderate free energy barrier of 14.8 kcal mol^–1^ (R15). The stability of dihydroxyacetone alkoxide anion (**16**) results in a rate-determining barrier of 20.6 kcal mol^–1^ for the subsequent enolization of dihydroxyacetone (**17**, R17; deprotonation at C-1). For the stability of trioses, while there is consensus that dihydroxyacetone (**17**) is slightly more stable than glyceraldehyde (**7**) in neutral aqueous solution,^31^ our calculations suggest that under formose reaction conditions, this stability order reverses due to interactions of the trioses with Ca^2+^ and OH^–^, as shown in Figure S4. This makes the conversion of glyceraldehyde (**7**) to the dihydroxyacetone enolate anion (**14**) slightly endothermic by 3.2 kcal mol^–1^.

Next, as a nucleophile, the dihydroxyacetone enolate anion (**14**) tends to attack formaldehyde to yield **18** (R18), which is then protonated to give ketotetrose (**19**, R19). The reaction steps are progressively exothermic, with a combined free energy release of 11.3 kcal mol^–1^. Similar to aldotriose-ketotriose tautomerization, the subsequent conversion of ketotetrose (**19**) to aldotetrose (**23**) may also proceed via two distinct pathways: the enolization pathway (pink) involves four sequential proton transfers through the C4 enol (**21**, R20–23), while the hydride-shift pathway (green) features a 1,2-hydride reverse migration (from C-1 to C-2; R25). They both exhibit moderate free energy barriers, i.e., 13.9 and 14.8 kcal mol^–1^, respectively (Figure 2c). The most critical steps in the Breslow autocatalytic cycle turn out to be the deprotonation and retroaldol cleavage of aldotetrose (**23**, R27–28), which regenerate glycolaldehyde (**4**) and its enolate anion (**5**), the precursors for cycle propagation. The cleavage process is endothermic, with overall barriers of 18.0 and 22.2 kcal mol^–1^ relative to the most stable intermediates along the enolization and hydride-shift pathways. Even so, the retroaldol cleavage pathways remain more favorable than formaldehyde dimerization (R1–3; *G*_a_ = 26.9 kcal mol^–1^, see Figure 1), demonstrating the role of Breslow autocatalytic cycle.

### 3.4 Beyond the Basic Network

The previous section outlined the foundational network for the formose reaction, with a focus on the linear C3–C5 sugars and the Breslow autocatalytic cycle. Yet, as experiments^15,20,21,24^ demonstrate, its full complexity is considerably greater, encompassing branched sugars and their associated catalytic cycles. Within the scope of this study, we offer only a preliminary discussion of these extended pathways, as a complementary perspective to the foundational network, leaving a complete analysis of these complex aspects to future work.

We commence with a brief discussion of an alternative autocatalytic cycle via branched pentose (see Figure S5), following the proposal of Benner et al.^15^ Setting aside the previously discussed enolization and aldol steps (**12** → **22**), we focus on the subsequent key step: the aldol addition of formaldehyde to aldotetrose enolate anion (**22**), which yields the branched pentose alkoxide anion (**27**) with an overall free energy barrier of 18.8 kcal mol^–1^ (R29). The anion then tautomerizes to **29** via proton exchange with H_2_O and OH^–^ (R30, 31), and subsequently undergoes retroaldol cleavage to reform glycolaldehyde (**4**) and glyceraldehyde enolate anion (**12**, R32). With an overall exothermicity of 4.6 kcal mol^–1^, this cycle appears thermodynamically favored over the Breslow cycle.

Next, we turn to a brief discussion of the other tetrose products. Our calculations show that the branched tetrose (**30**) has a relative free energy of −36.1 kcal mol^–1^ (Figure S5c), which is higher than that of the linear tetroses (−37.0 to −42.0 kcal mol^–1^, Figure 2c). As a dead-end product incapable of further enolization or aldol addition, the branched tetrose’s poorer stability rationalizes its relatively minor product formation in the long term formose experiments.^24^ By contrast, the furanose tetroses (**32** and **33**) exhibit comparable stability to that of the linear tetroses, with relative free energies of −41.1 and −38.3 kcal mol^–1^, respectively.

### 3.5 Microkinetic Simulation

Using the overall reaction network and free energy profiles, we have performed a microkinetic simulation to elucidate the kinetic details of the formose reaction, as illustrated in Figures 4, S6, and S7. The simulation involves formaldehyde dimerization, Breslow autocatalytic cycle, and ribose synthesis (see Figures 1 and 2), comprising 28 elementary steps and 28 distinct species (as detailed in Supporting Table S1). The simulation conditions (65 °C, 0.35 M CH_2_O, 0.05 M HOCH_2_CHO, and 0.06 M OH^–^) matches Benner’s^27^ and Breslow’s^28^ experimental conditions to probe into their debate on the autocatalytic cycle.

**Figure 4:**
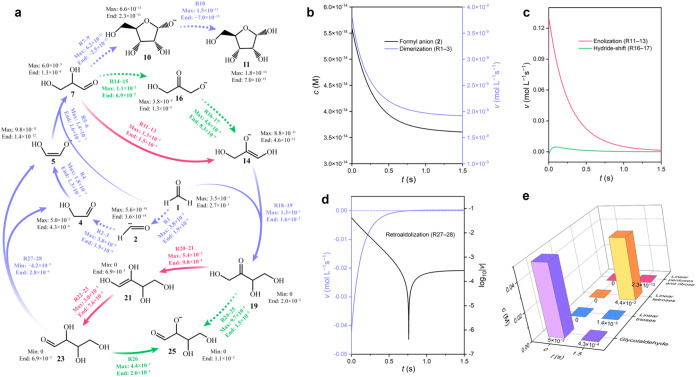
Microkinetic simulation of the formose reaction. (a) Overview of the microkinetic simulation, depicting critical species and their interconversions. The black labels indicate the concentration variation of the species during the simulation (in M, i.e., mol L^–1^), while the colorful labels represent the variation of reaction rates for the conversions (in mol L^–1^s^–1^): blue for the aldol/retroaldol reaction, pink for the enolization pathway, and green for the hydride-shift pathway. To better visualize the reaction progression, dominant pathways are denoted by solid arrows and minor pathways by dotted arrows. Although represented with unidirectional arrows, all conversions are kinetically reversible; the net direction is determined by the signs of the reaction rates. (b) Formyl anion (**2**) concentration (left axis) and formaldehyde dimerization rate (right axis) over simulation time. (c) Comparison of reaction rates between enolization and hydride-shift pathways in the conversion of glyceraldehyde (**7**) to the dihydroxyacetone enolate anion (**14**). (d) Retroaldolization rate of aldotetrose alkoxide anion (**26**, left axis) and its logarithmic absolute value (right axis) over simulation time. (e) Concentration variation of the C2–C5 species during the simulation.

Based on the simulation results, we now begin to analyze the kinetic details. For the initial formaldehyde (**1**) dimerization to glycolaldehyde (**4**) that accounts for long induction periods in experiments without initiators, our simulation reveals substantial kinetic inhibition of this conversion (Figure 4a, dotted arrows). The detailed plot in Figure 4b shows that the key intermediate, formyl anion (**2**, CHO^–^), maintains an extremely low concentration (5.6 × 10^–14^ → 3.6 × 10^–14^ M) during the simulation, resulting in its low coupling rate with formaldehyde (3.8 × 10^–9^ → 1.9 × 10^–9^ mol L^–1^s^–1^). This is consistent with the considerable endothermicity (15.2 kcal mol^–1^) for formyl anion formation and the large overall free energy barrier (26.9 kcal mol^–1^) for formaldehyde dimerization (Figure 1). Without alternative reaction channels, the formose reaction would be kinetically disfavored from the outset.

To bypass the trivial induction period, a certain amount of glycolaldehyde (**4**, 0.05 M) is introduced as feedstock in the simulation to accelerate the reaction, mimicking glycolaldehyde-initiated experimental conditions. With moderate free energy barriers (10–20 kcal mol^–1^) for both the aldol reaction and aldose-ketose tautomerization (Figures 1 and 2), the system achieves substantial conversion in early stages. Since two competing pathways, enolization^25^ and hydride-shift^28,50^ (Figure 2), have been proposed for aldose-ketose tautomerization, their reaction rates are plotted as a function of time in Figure 4c for comparison. Evidently, the enolization pathway dominates the conversion, although its reaction rate decreases over time as glycolaldehyde (**4**) is consumed. The simulation results are consistent with the distinct free energy barriers of 12.6 and 20.6 kcal mol^–1^ for the enolization and hydride-shift pathways, respectively.

Next, for the Breslow autocatalytic cycle, the pivotal step is the retroaldol cleavage of the aldotetrose alkoxide anion (**26**, R28), which produces glycolaldehyde (**4**) and its enolate anion (**5**), enabling cycle propagation. To investigate this simplest autocatalytic cycle mechanism, Figure 4d depicts the reaction rate for the pivotal step, along with the logarithm of its magnitude. Importantly, the net reaction rate appears negative during initial stages, indicating dominance of the reverse aldol condensation of glycolaldehyde (**4**) that forms aldotetrose alkoxide anion (**26**). This is consistent with recent experimental observations of glycolaldehyde-initiated formose reactions.^23^ During this phase, the Breslow autocatalytic cycle is suppressed by the high glycolaldehyde concentration, which favors aldol condensation. Nevertheless, as glycolaldehyde (**4**) is depleted, the net reaction rate turns positive at 0.76 s, indicating that the retroaldol cleavage of the aldotetrose alkoxide anion (**26**) becomes dominant. The cleavage rate, however, reaches only 2.8 × 10^–4^ mol L^–1^s^–1^ by the end of the simulation, 2 orders of magnitude lower than the initial condensation rate of –4.2 × 10^–2^ mol L^–1^s^–1^. This suggests that the Breslow autocatalytic cycle operates exclusively at low glycolaldehyde concentrations and exhibits limited catalytic efficiency, thereby posing a detection challenge in initiator-facilitated formose experiments.

Finally, Figure 4e presents the aggregate raw material consumption and product formation from the microkinetic simulation. Importantly, the scope of this simulated flow, as defined by our calculated reaction network (Figures 1 and 2), is limited to formaldehyde, glycolaldehyde, linear C3–C5 sugars, as well as ribose. Within this scope, the simulation shows a key stability reversal: consumed formaldehyde and glycolaldehyde are channeled predominantly into linear tetroses, rather than into linear pentoses and ribose. This reversal is likely due to the weaker Ca^2+^ complexation of longer-chain sugars, which offers a mechanistic rationale for the experimental low yield of ribose.^5^ While our model successfully captures the kinetics within its scope, the more complex chemistry responsible for the experimentally identified branched and condensed (C6–C9) sugars^15,20,21,24,27^ remains a subject for future theoretical investigation.

## 4 Conclusions and Outlook

The chemical research community continues to face challenges in elucidating the underlying mechanisms of complex reaction processes, a task that depends critically on *in situ* experimental detection and analysis. The formose condensation represents an important model system that has prompted numerous experimental studies.^15,20,21,23,24,27,28,50^ By contrast, existing theoretical calculations cannot adequately elucidate such intricate reaction mechanisms.^31,32,35,37^ The limitation stems from conventional computational methods, such as TS search^40–42^ and enhanced sampling,^51,52^ relying on preset reaction coordinates, which constrains their predictive power.

In recent years, several novel approaches have been proposed to address this limitation, such as *ab initio* nanoreactor (AINR),^9^ stochastic surface walking (SSW) sampling,^53^ and yet another reaction program (YARP).^54^ Building on these developments, we establish a computationally efficient approach that integrates our RTIP method^13^ with conventional MD for reaction simulations. Derived from pristine Cartesian coordinates, the RTIP eliminates constraints on bond lengths and angles, permitting mechanism-free simulations. Based on RTIP-MD, we have mapped a comprehensive reaction network and performed a microkinetic simulation for the formose reaction, clarifying the long-standing questions regarding formaldehyde self-condensation, ribose synthesis, and the autocatalytic cycle. Specifically, we reveal that (i) the formyl anion forms under alkaline conditions, inducing umpolung that enables nucleophilic attack on the carbonyl carbon of a second formaldehyde molecule, leading to the puzzling formaldehyde self-condensation to glycolaldehyde; (ii) the ribose synthesis can proceed via C3 + C2 → C5 coupling, bypassing tetrose formation; (iii) aldose-ketose tautomerization occurs predominantly via the enolization pathway, with only minor hydride-shift participation; (iv) as the pivotal step in the Breslow autocatalytic cycle, the aldotetrose retroaldol cleavage operates exclusively at low glycolaldehyde concentrations, with limited catalytic efficiency. These theoretical insights provide a foundation for designing tailored experiments to investigate specific reaction mechanisms, including: (i) detecting the microscale formyl anion to validate our proposed formaldehyde self-condensation mechanism and (ii) using isotope labeling to track the retroaldol cleavage of aldotetrose at low glycolaldehyde concentrations to examine the Breslow autocatalytic cycle mechanism.

While our RTIP-MD approach has proven effective for organic reactions, we are now extending this method to enzyme-catalyzed systems, which contain tens of thousands of atoms and feature structurally heterogeneous active center environments. This presents new methodological challenges, particularly in designing a precisely tuned RTIP that accelerates the reaction while preserving the enzyme’s delicate active center architecture. We expect the method’s adaptability across diverse reaction environments and scalability for large-scale molecular simulations.

## Supplementary Material

















## Data Availability

All original code has been deposited at https://github.com/MillenniumDream/RTIP-MD as of the date of publication.

## Notes

RTIP methodology; TS structures of all steps in the reaction network; geometric structures of all species in the reaction network; comparative stability of glyceraldehyde (**7**) and dihydroxyacetone (**17**); mechanistic details on branched and furanose sugars; concentrations of all species over simulation time in the microkinetics; reaction rates of all steps over simulation time in the microkinetics; Gibbs free energy profiles for all reaction steps; coordinates of all species and TS structures in the reaction network (PDF)
Synthesis of glyceraldehyde from formaldehyde (Video 1) (MP4)
Synthesis of linear pentose from glyceraldehyde (Video 2) (MP4)
Synthesis of ribose from linear pentose (Video 3) (MP4)
Isomerization of glyceraldehyde via enolization (Video 4) (MP4)
Isomerization of glyceraldehyde via hydride-shift (Video 5) (MP4)
Isomerization of glyceraldehyde via proton transfer (Video 6) (MP4)
Retroaldol cleavage of aldotetrose (Video 7) (MP4)
