# Supplementary material for: Conserved-Potential-Driven Molecular Dynamics Deciphers Formose Reaction Mechanisms
Source: JACS Au. 2026 Jan 22;6(2):922–31. doi: 10.1021/jacsau.5c01359 (PMC12933356; doi:10.1021/jacsau.5c01359)
Supplement: Supplementary file 1 [file au5c01359_si_001.pdf]

Supporting Information for

Conserved-Potential-Driven Molecular Dynamics Deciphers Formose Reaction Mechanisms

Hei Wun Kan,[1†] Xiao-Tian Li,[1†*] Tong Zhu,[2,3] Yuzhi Xu,[3,4] John Zeng Hui Zhang[1,3,4,5*]

[1]Faculty of Synthetic Biology, Shenzhen University of Advanced Technology, Shenzhen 518107, China

[2]Shanghai Engineering Research Center of Molecular Therapeutics and New Drug Development, School of Chemistry and Molecular Engineering, East China Normal University, Shanghai 200062, China

[3]NYU-ECNU Center for Computational Chemistry, NYU Shanghai, Shanghai 200126, China

[4]Department of Chemistry, New York University, New York 10003, United States

[5]Collaborative Innovation Center of Extreme Optics, Shanxi University, Taiyuan 030006, China

[†]These authors contributed equally to the theoretical calculations and should be regarded as co-first authors

*Correspondence: lixiaotian@suat-sz.edu.cn

*Correspondence: john.zhang@nyu.edu

RTIP METHODOLOGY

Roto-translationally Invariant Potential

In chemical space exploration or molecular dynamics simulation, a faithful representation of the atomic interactions is crucial for preserving the system's symmetry, as governed by the physical conservation laws. While the atomic potentials, ranging from quantum chemistry methods, semi-empirical models, and classical force fields, naturally comply with the symmetry constraints as a result of their rigorous theoretical foundations, the virtual potentials potentially face challenges due to their directional specificity for enhanced sampling. Traditionally, certain roto-translational invariants like interatomic distances and angles are chosen to construct the representation space for the virtual potential.[1-3] Such representation spaces, however, appear tedious, demanding meticulous attention to the bond dissociation and reassociation, which limits the predictive capability of the virtual potential.[4] Here, we present a general potential that preserves rotational and translational symmetry as formulated with Cartesian generalized coordinates, namely roto-translationally invariant potential (RTIP), for enhanced sampling.

In three-dimensional Euclidean space, the geometric configuration of a molecular system consisting of n atoms can be represented by tuples of Cartesian coordinates for each atom, denoted as $\mathbf{R} = (\mathbf{r}_1,\cdots,\mathbf{r}_n)$, where $\mathbf{r}_i = (x_i,y_i,z_i)^{\mathrm{T}} \in \mathbb{R}^3$. For refined representation of spatial transformation, atomic homogeneous coordinates, expressed as $\mathbf{p}_i = (x_i,y_i,z_i,1)^{\mathrm{T}}$, could be introduced as a substitute for Cartesian coordinates. In this formalism, the roto-translation operation applied to a molecular system $\mathbf{P} = (\mathbf{p}_1,\cdots,\mathbf{p}_n)$ is faithfully represented by a 4×4 matrix

$$\mathbf{S} = \begin{pmatrix} \mathbf{R} & \mathbf{t} \\ \mathbf{0} & 1 \end{pmatrix} \tag{1}$$

where $\mathbf{R}$ is the 3×3 rotation matrix and $\mathbf{t} \in \mathbb{R}^3$ is the translation vector. A metric between two molecular systems $\mathbf{P}_1$ and $\mathbf{P}_2$ can be defined as

$$d_{\mathbf{P}_1,\mathbf{P}_2} := \min_{\mathbf{S}} ||\mathbf{P}_1 - \mathbf{S}\mathbf{P}_2|| \tag{2}$$

where $||\,||$ symbolizes the Frobenius norm. It is worth noting that, this metric manifests roto-translational invariance with respect to both molecular systems, as a consequence of their proper spatial alignment before evaluation of the Frobenius norm (i.e. finding the optimal $\mathbf{S}$ that acts on $\mathbf{P}_2$ for the minimum norm). More importantly, functions derived from this metric likewise demonstrate roto-translational invariance, providing a generalized formulation for the symmetry-conserved virtual potential.

In practical implementations, the optimal translation vector $\mathbf{t}$ can be easily determined by aligning the centroids of $\mathbf{P}_1$ and $\mathbf{P}_2$, whereas the optimal rotation matrix $\mathbf{R}$ can be ascertained using the the quaternion method.[5] In quaternion formalism, the least-square fitting of $\mathbf{P}_1$ and $\mathbf{P}_2$ is represented by a 4×4 real symmetric matrix, whose spectral factorization yields four eigenvalues, $\lambda_1 \leq \lambda_2 \leq \lambda_3 \leq \lambda_4$, along with their corresponding eigenvectors: $\boldsymbol{\alpha}_1$, $\boldsymbol{\alpha}_2$, $\boldsymbol{\alpha}_3$, $\boldsymbol{\alpha}_4$ (see Figure S1a). The optimal rotation matrix $\mathbf{R}$ then can be derived from the eigenvector $\boldsymbol{\alpha}_1$, which is associated with the minimum eigenvalue λ_1.

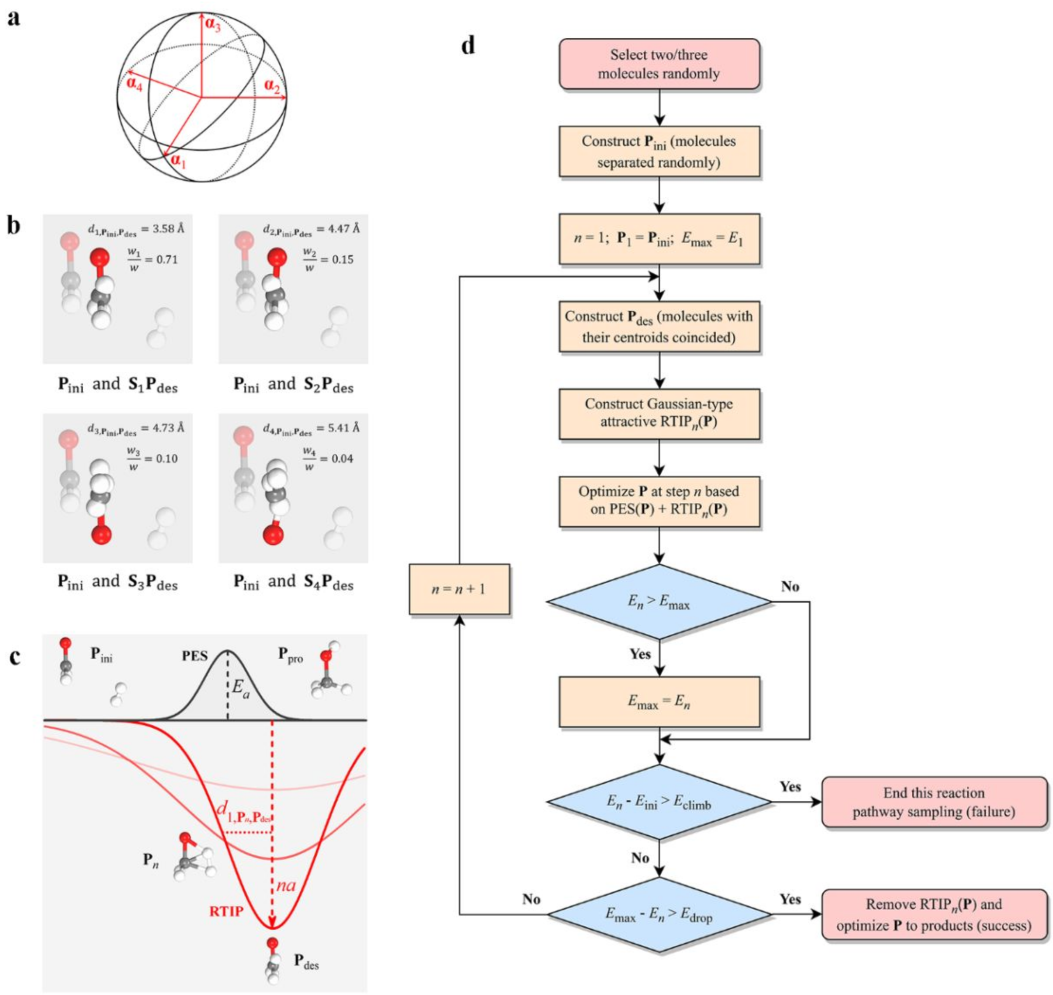

Figure S1. Schematic diagram of RTIP methodology. (**a**) Least-square fitting of two configurations in quaternion representation, with four orthogonal eigenvectors resolved by spectral factorization. (**b**) Four corresponding alignment modes between the initial configuration $\mathbf{P}_{\text{ini}}$ (the separated formaldehyde and hydrogen gas in a semitransparent form) and the destination configuration $\mathbf{P}_{\text{des}}$ (the coincided formaldehyde and hydrogen gas in an opaque form) in the direct hydrogenation of formaldehyde. (**c**) Schematic diagram for the real PES and the attractive RTIP Gaussian function of $d_{1,\mathbf{P},\mathbf{P}_{\text{des}}}$, whose depth increases linearly over the searching steps. (**d**) Flow chart of the RTIP pathway sampling.

In fact, the four eigenvectors delineate four distinct rotation operations, each correlating to a unique alignment mode upon combination with the optimal translation vector **t**. Significantly, across all the four alignment modes, the difference vector ($\mathbf{P}_1$–$\mathbf{SP}_2$) exhibits inherent orthogonality to the generators of SO(3) group operating on both $\mathbf{P}_1$ and $\mathbf{P}_2$. This suggests that the four associated Frobenius norms, denoted as $d_{i,\mathbf{P}_1,\mathbf{P}_2}$ where $i = 1, 2, 3, 4$, are intrinsically roto-translationally invariant, rendering them appropriate for the construction of virtual potential. To tackle the non-differentiability issue arising from degenerate eigenvalues, the RTIP is systematically formulated in a combination manner as follows:

$$w_i = \frac{1}{d^7_{i,\mathbf{P}_1,\mathbf{P}_2}},\ i = 1, 2, 3, 4 \tag{3}$$

$$w = \sum_{i=1}^{4} w_i \tag{4}$$

$$\mathrm{RTIP}_{\mathbf{P}_1,\mathbf{P}_2} = \sum_{i=1}^{4} \frac{w_i}{w} f(d_{i,\mathbf{P}_1,\mathbf{P}_2}) \tag{5}$$

Herein, the general schema of RTIP bridging $\mathbf{P}_1$ and $\mathbf{P}_2$ is defined as the weighted average across a function of $d_{i,\mathbf{P}_1,\mathbf{P}_2}$. When $\lambda_1 \ll \lambda_2 \ll \lambda_3 \ll \lambda_4$, $\mathrm{RTIP}_{\mathbf{P}_1,\mathbf{P}_2}$ is primarily governed by $d_{1,\mathbf{P}_1,\mathbf{P}_2}$, corresponding to the optimal alignment mode. While in degenerate cases, e.g. $\lambda_1 = \lambda_2 \leq \lambda_3 \leq \lambda_4$, $\mathrm{RTIP}_{\mathbf{P}_1,\mathbf{P}_2}$ is equally impacted by both $d_{1,\mathbf{P}_1,\mathbf{P}_2}$ and $d_{2,\mathbf{P}_1,\mathbf{P}_2}$, thereby preventing a discontinuous transition from λ_1 to λ_2.

Gaussian-type Attractive RTIP

Within the combination framework of RTIP, we now introduce its application for probing the reactivity of organic molecules. As depicted in the flow chart in Figure S1d, the pathway sampling is commenced with the random selection of either two or three molecules from the candidate list, allowing for repetition if necessary. Subsequently, the selected molecules are randomly placed in a distance from the centroid (a 5 Å separation is adopted, since the size of the molecules involved is smaller than 5 Å), establishing the initial configuration $\mathbf{P}_{\mathrm{ini}}$ (as exemplified by the separated formaldehyde and hydrogen gas in Figure S1c). To investigate the reactivity of the molecules, a virtual structure is deliberately fabricated as the destination configuration $\mathbf{P}_{\mathrm{des}}$, with the centroids of the molecules coincided (as illustrated by the overlapped formaldehyde and hydrogen gas in Figure S1c). Through spectral factorization, four alignment modes between $\mathbf{P}_{\mathrm{ini}}$ and $\mathbf{P}_{\mathrm{des}}$ can be determined (please refer to the detailed structures in Figure S1b), based on which a Gaussian-type attractive RTIP increasing over steps is defined as

$$\mathrm{RTIP}_n(\mathbf{P}) = \sum_{i=1}^{4} -\frac{w_i}{w} \cdot (na) \cdot \exp\left(-\frac{d_{i,\mathbf{P},\mathbf{P}_{\mathrm{des}}}^2}{2d_{i,\mathbf{P}_n,\mathbf{P}_{\mathrm{des}}}^2}\right) \tag{6}$$

where n denotes the searching step, a is the initial depth of the Gaussian function, and $\mathbf{P}_n$ is the current configuration at step n.

In practical implementation, the RTIP is superimposed onto the real potential energy surface (PES) to enable pathway sampling, as depicted in Figure S1c. During the iterative optimization, the attractive RTIP Gaussian functions progressively increase, pushing the molecules towards closer for potential reactions, while the real PES applied on the molecules determines their ultimate reactivity. Throughout the optimization process, two crucial parameters are monitored at every iteration to ascertain the cut-off point. The first parameter, denoted as (E_n − E_{ini}), is the energy difference between the current configuration (at step n) and the initial configuration on the real PES. This parameter must remain below the predefined threshold E_{climb}, as we aim to explore the low-energy reactions with $E_{\mathrm{a}} < E_{\mathrm{climb}}$. Otherwise, it suggests that the molecules are either unreactive or misaligned for the potential reaction, resulting in a failed pathway sampling. The second parameter, represented as (E_{max} − E_n), corresponds to the energy difference between the maximum-energy configuration (within the present pathway sampling) and the current configuration (at step n) on the real PES. When this parameter surpasses the specified threshold E_{drop}, it signifies the cleavage of preexisting chemical bonds and the subsequent formation of new bonds. Since the barrier has been overcome, it is imperative to promptly eliminate the RTIP, which is destined for the virtual $\mathbf{P}_{\mathrm{des}}$. Thereafter, a local optimization is carried on based on the real PES, driving the current configuration $\mathbf{P}_n$ towards the desired products $\mathbf{P}_{\mathrm{pro}}$. In summary, the predefined thresholds E_{climb} and E_{drop} serve as the convergence criteria for the failed and successful pathway sampling, respectively: the former specifies the highest barrier in the search, while the latter refers to the release of energy for confirming the formation of new chemical bonds.

Program Implementation

The RTIP method has been implemented using the Rust programming language, which is available online under the MIT or Apache-2.0 License (https://github.com/MillenniumDream/RTIP). As the pathway samplings are independent from each other, they can expediently perform in parallel. Besides, the RTIP program has been linked to the CP2K package, which can provide a range of PES, involving xTB, DFT, quantum mechanics/molecular mechanics (QM/MM), for the pathway sampling. This enables the method to deal with more complicated simulations, e.g. including solvent effect via the CP2K input, or considering the long-range interaction in a QM/MM simulation.

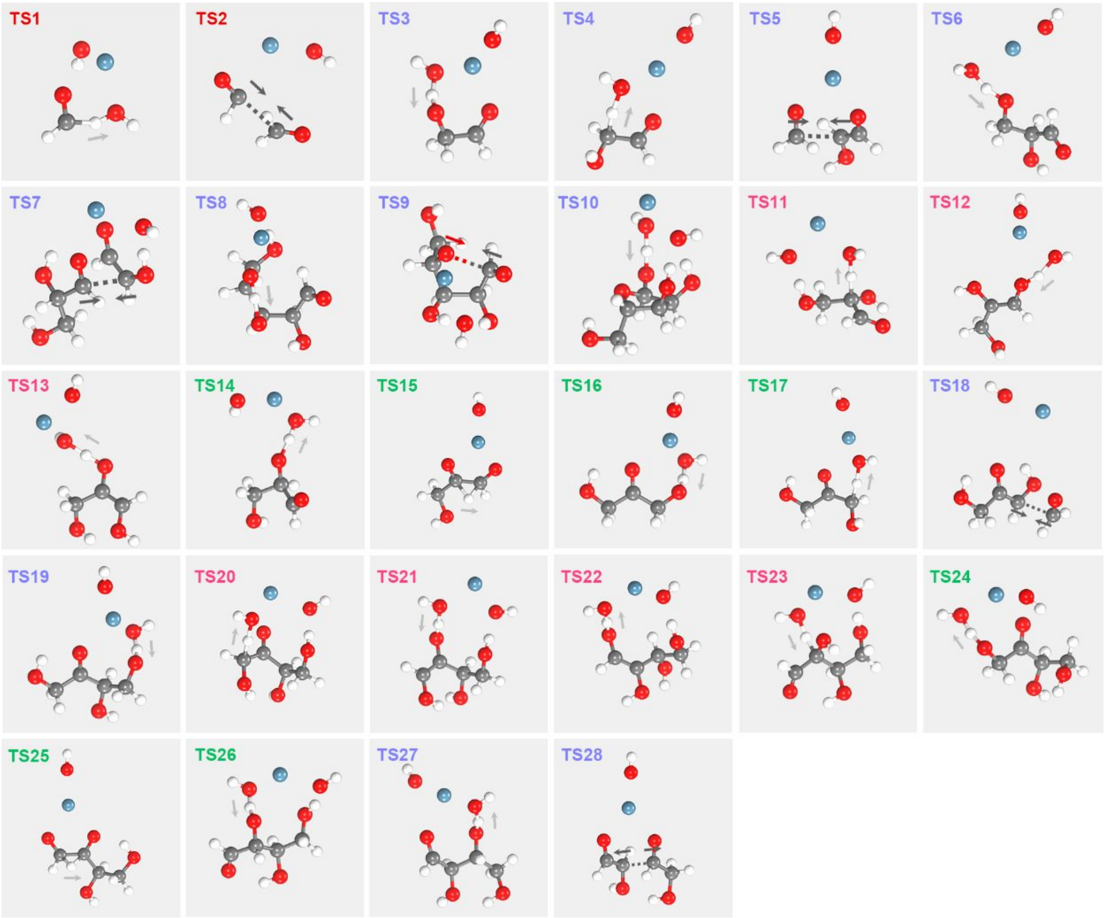

Figure S2. Transition state (TS) structures of all steps in the reaction network. The arrows depict the imaginary-frequency vibrational mode at TS, illustrating the atomic displacements along the reaction coordinate.

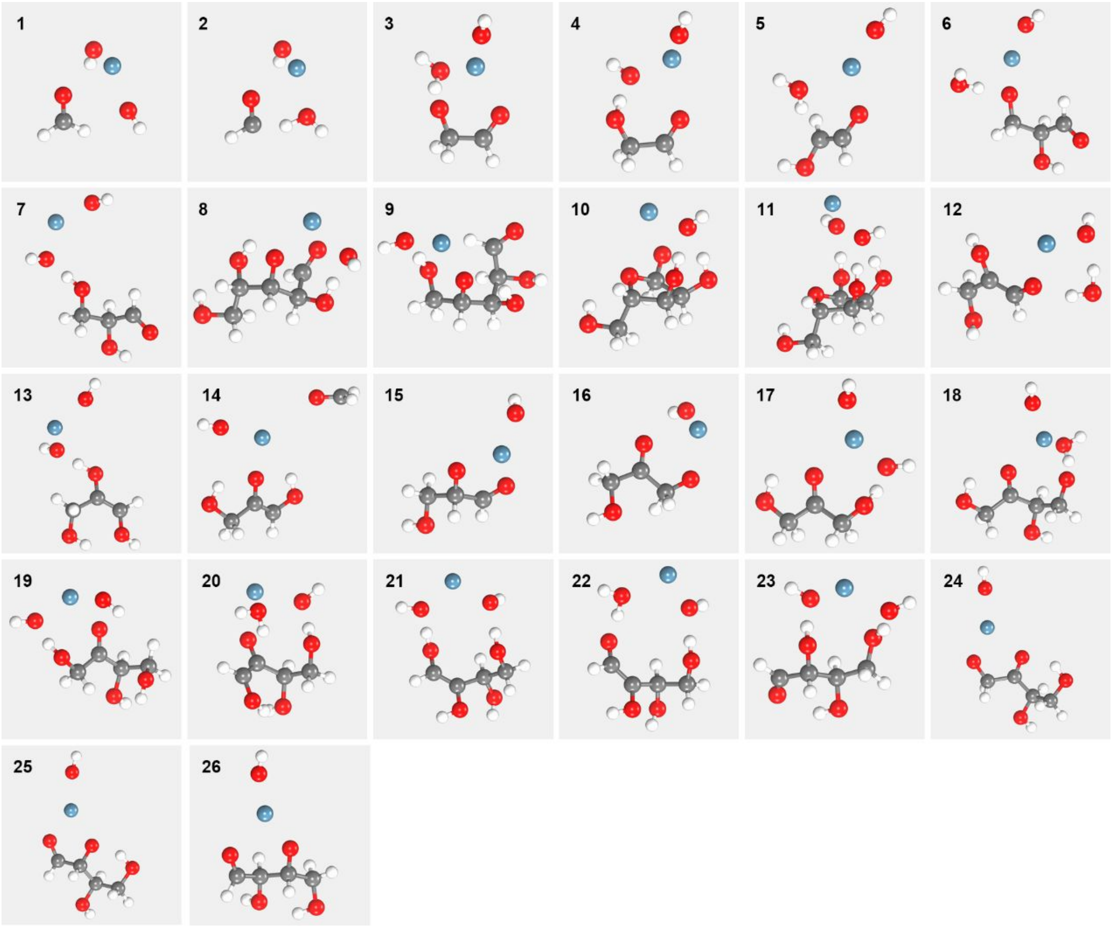

Figure S3. Geometric structures of all species in the reaction network.

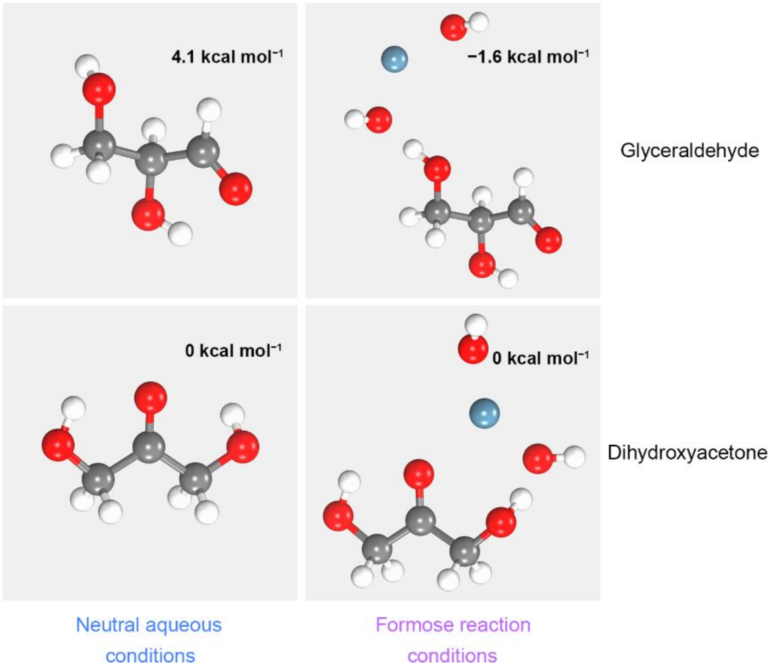

Figure S4. Comparative stability of glyceraldehyde (7) and dihydroxyacetone (17) under neutral versus alkaline (formose) aqueous conditions, with dihydroxyacetone serving as the baseline.

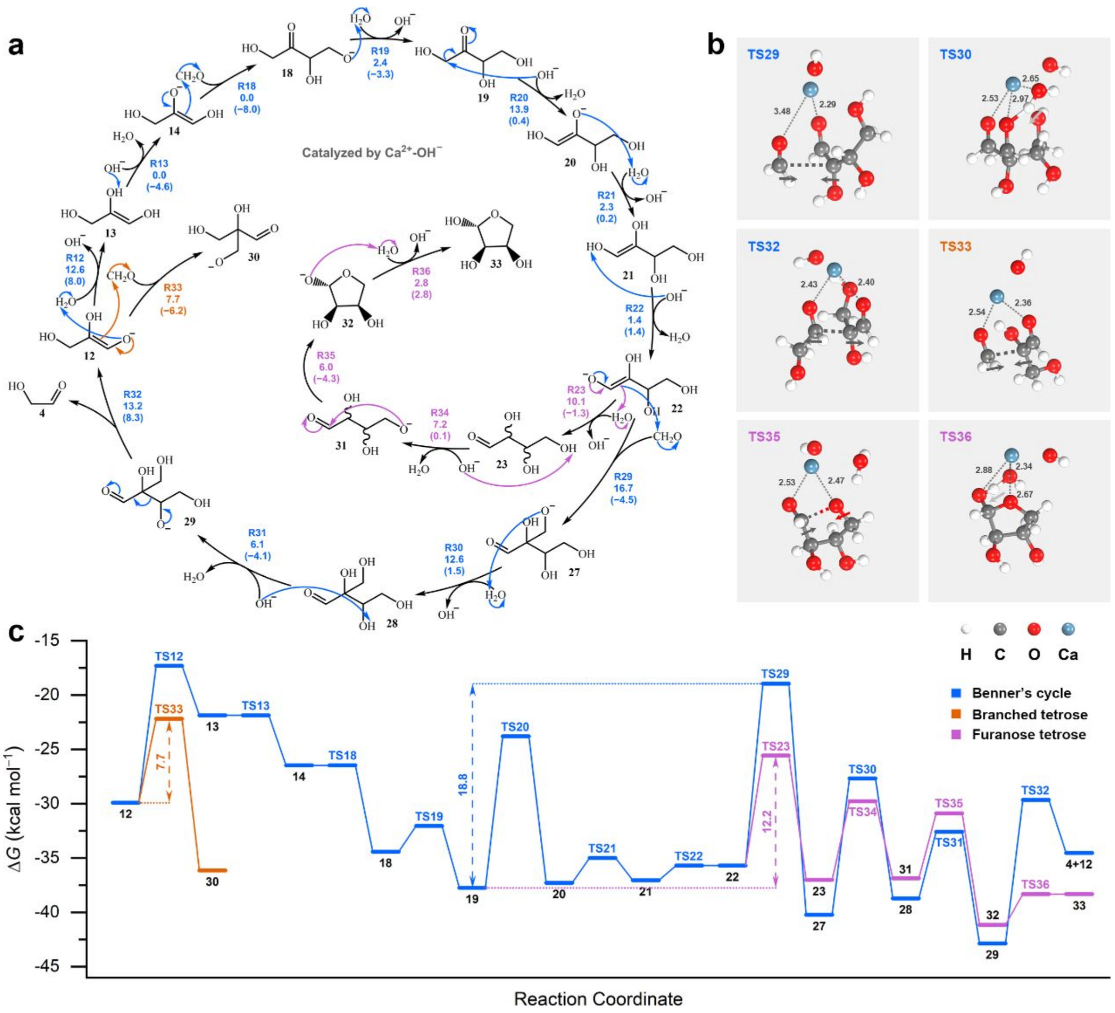

Figure S5. Mechanistic details on branched and furanose sugars in the formose reaction. (a) Stepwise reaction network with the Benner autocatalytic cycle (blue), and the synthetic pathways to branched (yellow) and furanose (purple) tetroses highlighted in different colors. (b) TS structures for the key steps selected from the reaction network. (c) Gibbs free energy profiles (at 55 °C, 1atm, 1 mol L^{-1}) for the reaction network.

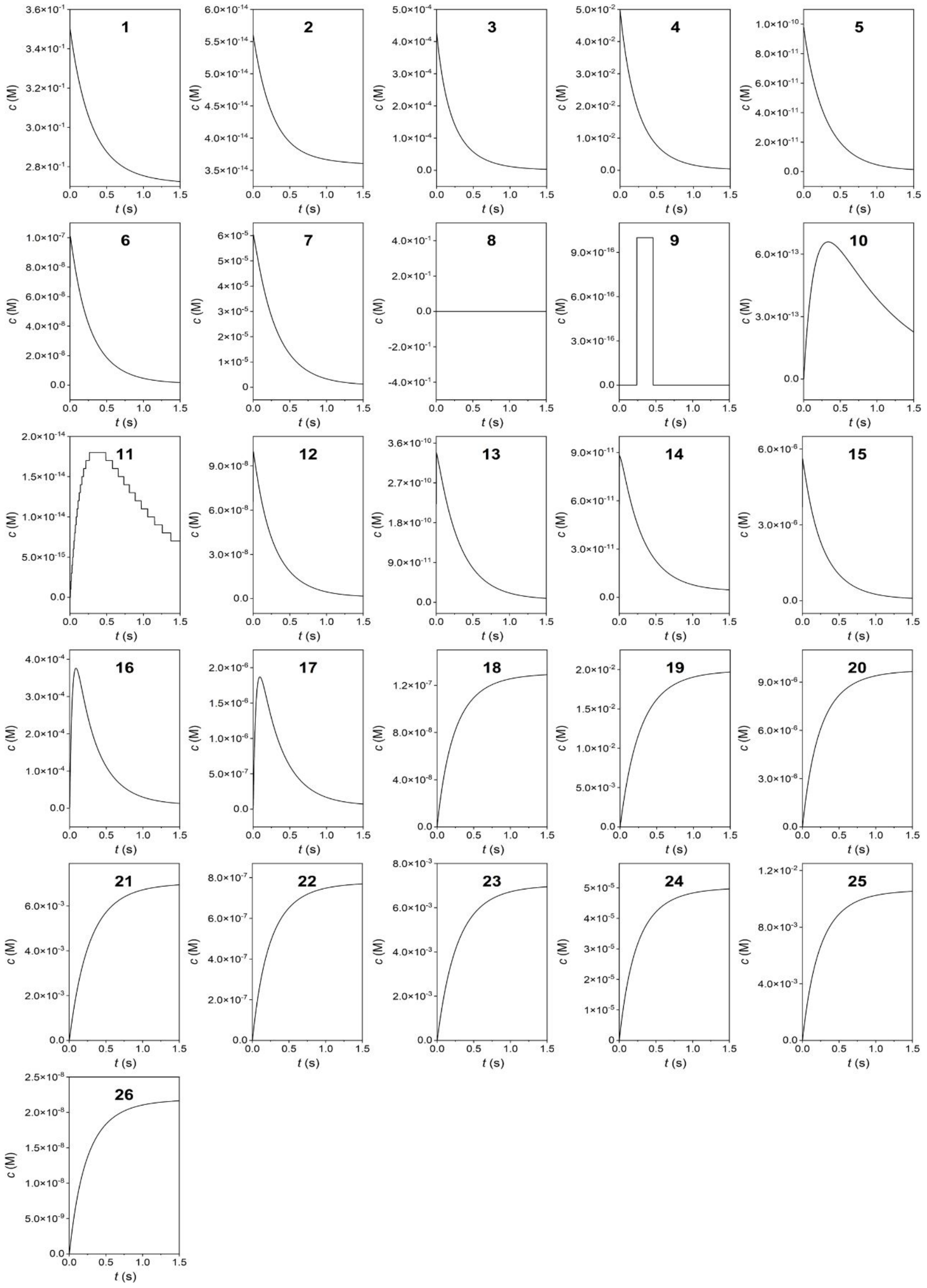

Figure S6. Concentrations of all species over simulation time in the microkinetics.

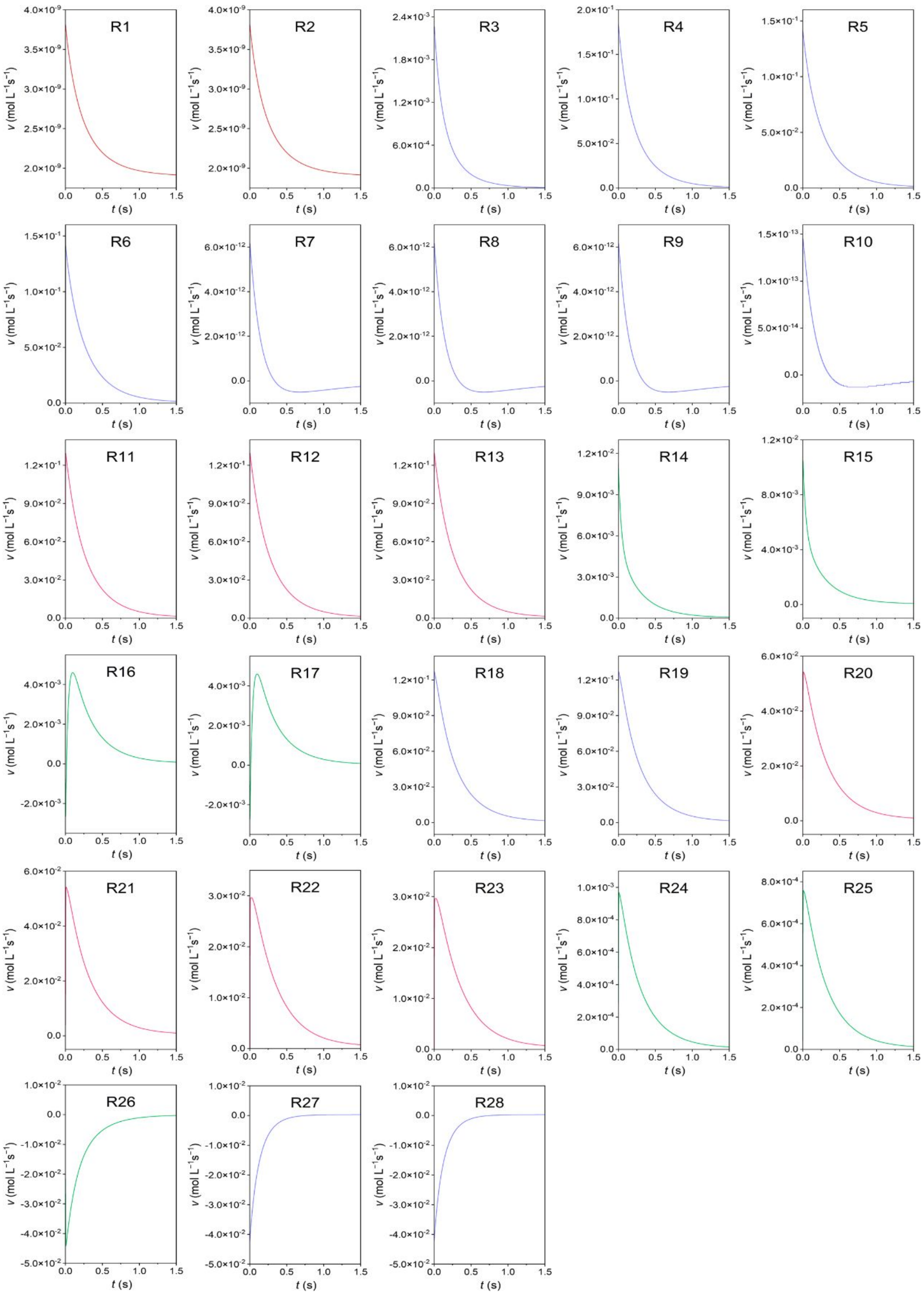

Figure S7. Reaction rates of all steps over simulation time in the microkinetics.

Table S1. Gibbs free energy profiles (in kcal mol^{-1}, at 55 °C, 1 mol L^{-1}) for all reaction steps in Figures 2 and 3. Of note, to achieve a large timestep (5.0×10^{-12} s) for the efficient simulation, most of the low barriers are elevated to 5.0 kcal mol^{-1} with the exothermicity/endothermicity remaining constant, which would have little effect on the reaction kinetics. The simulation was performed at 65 °C with the following initial concentrations: $c(CH_2O)$ = 0.35 M, $c(HOCH_2CHO)$ = 0.05 M, $c(OH^-)$ = 0.06 M, $c(H_2O)$ = 55.5 M, with all other species concentrations set to zero.

Index	Chemical equation	Forward barrier	Reverse barrier
1	**1** + OH^- ⇄ **2** + H_2O	20.2	5.0
2	**2** + **1** ⇄ **3**	11.7	48.0
3	**3** + H_2O ⇄ **4** + OH^-	6.4	5.0
4	**4** + OH^- ⇄ **5** + H_2O	17.1	12.0
5	**5** + **1** ⇄ **6**	5.0	20.3
6	**6** + H_2O ⇄ **7** + OH^-	5.3	5.0
7	**7** + **5** ⇄ **8**	15.2	12.7
8	**8** ⇄ **9**	7.0	9.3
9	**9** ⇄ **10**	5.0	9.8
10	**10** + H_2O ⇄ **11** + OH^-	12.1	5.0
11	**7** + OH^- ⇄ **12** + H_2O	9.9	10.2
12	**12** + H_2O ⇄ **13** + OH^-	12.6	4.6
13	**13** + OH^- ⇄ **14** + H_2O	4.5	9.1
14	**7** + OH^- ⇄ **15** + H_2O	5.7	8.7
15	**15** ⇄ **16**	14.8	18.4
16	**16** + H_2O ⇄ **17** + OH^-	13.2	5.0
17	**17** + OH^- ⇄ **14** + H_2O	12.4	10.9
18	**14** + **1** ⇄ **18**	5.0	13.0
19	**18** + H_2O ⇄ **19** + OH^-	5.0	8.3
20	**19** + OH^- ⇄ **20** + H_2O	13.9	13.5
21	**20** + H_2O ⇄ **21** + OH^-	5.3	5.0
22	**21** + OH^- ⇄ **22** + H_2O	6.4	5.0
23	**22** + H_2O ⇄ **23** + OH^-	10.1	11.5
24	**19** + OH^- ⇄ **24** + H_2O	5.0	5.7
25	**24** ⇄ **25**	14.8	18.4
26	**25** + H_2O ⇄ **23** + OH^-	10.0	5.0
27	**23** + OH^- ⇄ **26** + H_2O	8.8	5.0
28	**26** ⇄ **4** + **5**	13.5	4.5

Coordinates of all species and TS structures in the reaction network

1

9

i =	142, E =	-944.0077097540	
O	1.6512021776	4.9557743724	15.8709538645
C	2.2064456798	5.4630135241	16.8216632803
H	2.0589340241	6.5275316988	17.0487150920
H	2.8649207092	4.8507202247	17.4511963233
O	3.5645396075	2.8041248027	17.0879212225
H	4.3705484969	2.8408099313	17.5992496438
Ca	2.0947208269	2.3899333186	15.5405146139
O	0.2107662728	1.9442686881	16.6209245786
H	0.1375039789	2.1318277973	17.5592127135

2

9

i =	558, E =	-943.9827015983	
O	1.6394510800	5.0116182229	15.9062911153
C	2.2737732327	5.4996736250	16.8632694847
H	2.1093969549	6.6224151761	16.9370619542
H	3.0521451615	4.1987488209	17.5198590281
O	3.3093990469	3.1731987774	17.5725396080
H	4.2679122598	3.1378659634	17.6516928092
Ca	1.9504096948	2.5451675966	15.5955889631
O	0.2527760216	1.9617564735	16.8962991128
H	0.2403269921	2.1535312296	17.8363511767

3

13

i =	526, E =	-1058.5984460376	
O	0.2206686340	1.0078236017	0.1383791554
O	0.4338042821	-0.1463012779	2.5957582839
C	-0.3180441485	-0.2318657516	0.3317680459
C	-0.1401912737	-0.7660908011	1.7200641102
H	0.1037583290	-1.0259859203	-0.3216536763
H	-1.4086529798	-0.2914026817	0.1448336198
H	-0.5555608424	-1.7653981596	1.9335609330
H	1.8058569671	0.8086322403	0.0427972005
O	2.7601949401	0.8364422925	0.3644939892
H	3.2711608499	1.2785578116	-0.3213741711
Ca	1.0850935285	2.2568960986	1.9741797787
O	0.7553202365	4.2909618216	2.5323594577
H	0.5876106708	5.1921166011	2.7975559367

4

13

i =	945, E =	-1058.5949228061	
O	0.1294375176	0.7072040467	-0.4023935545
O	0.3164605498	0.2030484851	2.3707172769
C	-0.2848395461	-0.4969815837	0.1391052457
C	-0.1340355743	-0.6381641543	1.6241972607
H	0.2234344962	-1.3777230135	-0.2920290462
H	-1.3529657412	-0.6595666833	-0.0586531570
H	-0.4774206081	-1.6075348464	2.0293132061
H	1.1160380078	0.8783106143	-0.0964998126
O	2.4090627067	1.1845643205	0.5095384262
H	3.0932320751	1.3240059399	-0.1485864212
Ca	1.6568031825	2.4666227521	2.2663775838
O	0.7789969328	4.4217275567	2.5827946759
H	0.7319817626	5.3287899362	2.8778776485

5

13

i =	468, E =	-1058.5858480128	
O	1.8372087915	-0.1188883320	-0.1951904020
O	-1.7657599668	-0.1686435873	0.2503826847
C	0.5676809429	-0.6035059291	0.1718757172
C	-0.5342889846	0.1673391705	0.0189263748
H	-0.1418392672	-0.3895598748	2.1687626799
H	0.5165312425	-1.6610380228	0.4211806269
H	-0.3352273358	1.1979312996	-0.3229887501
H	2.3445547883	0.0380160180	0.6072846243
Ca	-3.0790913393	-0.6279710634	2.1166351061
O	-5.1190295953	-0.7619079404	2.7998187677
H	-6.0514991981	-0.9620616061	2.8429508826
O	-0.7190632947	-0.4314741322	2.9575401989
H	-0.2354508315	-0.9279865083	3.6261285653

6

17

i =	378, E =	-1173.1671941950	
O	-0.1067162613	1.5685200170	0.1692075501
O	1.9659441397	-1.0744926593	-1.2548331035
O	-1.6264562702	-0.5037505019	0.7953091086
C	0.1523840373	0.3619063064	-0.5142691580
C	1.6244250206	-0.0909819406	-0.3564236053
C	-0.7592928342	-0.7195902503	-0.0247972823
H	-0.0301727660	0.4790328978	-1.5906568456

H	1.7556850693	-0.4078586824	0.6971388262
H	2.2216039049	0.8327506275	-0.4590911331
H	-0.5817144902	-1.7261110978	-0.4281273839
H	-0.8693216828	1.4006860762	0.7445208078
Ca	1.9422784724	-1.5498790236	-3.5063379584
O	4.5368274316	-1.5952639584	-0.7543665262
H	4.6056308383	-2.4804802213	-0.3850868118
H	3.5599037767	-1.4453424502	-0.9061558268
O	1.6567001375	-3.7036219054	-3.4648839518
H	1.1918619502	-4.5121425313	-3.2564755322

7

17

i = 588, E = -1173.1652814506

O	-0.0267332174	1.6634101706	-0.0174876661
O	1.9106811841	-1.4325703939	-0.0734677641
O	-1.7288587622	-0.1900066344	0.8017215676
C	0.2710621693	0.3301243395	-0.3615770089
C	1.6459272629	-0.0874723466	0.1918231120
C	-0.7885536850	-0.6001107776	0.1566571661
H	0.2906959598	0.2113757540	-1.4532520178
H	1.6338579476	0.1100085193	1.2722238325
H	2.3955080866	0.5787196180	-0.2503735988
H	-0.6609436251	-1.6658638643	-0.0695738247
H	-0.8303747024	1.6331584539	0.5259464718
Ca	3.5303370592	-2.4529503473	-3.8766573973
O	3.7968735879	-1.6841864746	-1.7223642851
H	4.5563233587	-1.9250928979	-1.1852341385
H	2.6878929260	-1.5165531297	-0.7584277894
O	2.0238211465	-4.0184181516	-3.7768230206
H	1.3629910496	-4.6128894615	-3.4257558380

8

22

i = 490, E = -1325.8575717810

O	0.8258367959	0.9080633414	0.6849935173
O	0.5912933645	-1.6361382741	0.1751987353
O	-2.0403513307	-2.3955353247	1.3729299263
O	-1.9733896754	-0.5622159355	-0.7942204652
O	1.2075230127	1.8599833333	3.3941223956
C	0.1487683767	-1.2682511567	1.4197582953
C	0.8707800763	0.0404479356	1.8158173583
C	-1.4127234060	-1.1471121121	1.4588013274
C	-1.7947993147	-0.2066282334	0.3537929464

C	0.3508039604	0.7812711217	3.0264516619
H	0.3682136204	-2.0176200496	2.2037930429
H	1.9184381592	-0.2238751586	2.0168994027
H	-1.7165146686	-0.7002869086	2.4117139731
H	-1.8648691124	0.8627006358	0.6123167959
H	0.3183813213	0.1046753163	3.8818031764
H	-0.6625027664	1.1544200015	2.8447497265
H	-1.8642151353	-2.8687849631	0.4837878731
H	1.2793549019	2.4389703860	2.6259316517
Ca	-0.2401771345	-2.3704249538	-1.8358322668
O	-1.7298212931	-3.7278380755	-0.8154727065
H	-1.6067926251	-4.6331782731	-0.5213269671
H	0.8810706158	0.2525215104	-0.0431114861

9

22

i =	621, E =	-1325.8610189570	
O	0.4973444087	0.5956331436	-0.1003735821
O	0.1264964978	-1.9549791802	-0.1228329825
O	-2.1904337145	-2.4501829050	1.2906221820
O	-2.8383942767	-0.5268432205	-0.5305955696
O	-0.6073427764	1.5892570785	2.4169294472
C	-0.0535531340	-1.3350781943	1.1473781804
C	0.7572319111	-0.0255615061	1.1023135927
C	-1.5649352605	-1.1837303742	1.3907550267
C	-2.1693899754	-0.1903111511	0.4231585914
C	0.5862606530	0.8446323401	2.3570648522
H	0.3267628311	-2.0125384575	1.9178034800
H	1.8109082171	-0.3755701613	1.1532556826
H	-1.7322204728	-0.8051968025	2.4017758735
H	-1.9714183101	0.8600946172	0.6606303345
H	1.4537261134	1.5163542513	2.4178551269
H	0.6327470545	0.1859907111	3.2354758811
H	-1.9614823997	-2.7836139510	0.4115229469
H	-0.4809266233	2.3747511479	1.7823106216
Ca	0.1566828484	2.5525388816	-1.2042963011
O	-0.2243116717	3.5734191796	0.7953575567
H	0.3474706409	4.1818288685	1.2697725369
H	0.2557309965	-1.1515188546	-0.6789300509

10

22

i =	568, E =	-1325.8763752679	
O	0.4050414673	0.1663643849	0.9311409942

O	-0.1508918698	-2.6856349904	1.8517729919
O	-2.2770542760	-1.9324352307	0.2644370310
O	-0.8356676994	0.0465621849	-1.0082087283
O	0.7293185501	1.9104529914	3.0658189907
C	-0.7569409580	-1.4541751959	2.1739785063
C	0.2700250231	-0.3278678260	2.2559799426
C	-1.7435751249	-0.9272533453	1.0845241737
C	-0.9565670956	0.1915540203	0.2961949436
C	-0.1405864876	0.7947404611	3.2007707043
H	-1.2657797975	-1.5888245395	3.1343803800
H	1.2397109259	-0.7219297757	2.5641612268
H	-2.5870845757	-0.4509836075	1.5952389094
H	-1.3786855962	1.1732226105	0.5959717924
H	-0.0809439189	0.4463269782	4.2324663660
H	-1.1769973766	1.0969864870	3.0099241069
H	-1.5226337455	-2.2801168124	-0.2794934459
H	0.8641597416	2.0167011950	2.1137632159
Ca	1.0715209234	-1.0448240084	-1.7063637810
O	0.0232335144	-2.8666256044	-0.7888029918
H	0.0116913215	-3.8049988603	-0.9911328136
H	-0.0327700619	-2.7551570574	0.8591263465

11

25

i = 171, E = -1402.3303989343

O	0.0119507211	0.5597146596	0.5338290747
O	-0.1298045174	-2.5730660650	1.3137340234
O	-2.4199868932	-1.6647176824	0.0515599662
O	-1.8468320080	0.9317714156	-0.7849875712
O	1.0486728836	1.8876233895	2.7490343808
C	-0.6405881712	-1.3537195630	1.7869105779
C	0.3584645647	-0.1934043007	1.6942959100
C	-1.8906843055	-0.8012290677	1.0154583599
C	-1.4312652512	0.5845215683	0.4640561794
C	0.3040992493	0.6928329944	2.9325670293
H	-0.8984300790	-1.5168892015	2.8379420156
H	1.3718204186	-0.5679254570	1.5499886889
H	-2.6914530798	-0.6180552137	1.7350462805
H	-1.7867639973	1.3649295810	1.1546857327
H	0.7361434474	0.1546311420	3.7775597552
H	-0.7386925710	0.9232288929	3.1869926615
H	-1.7002686339	-2.0062419906	-0.5485467020
H	0.8150370970	2.2146336857	1.8693929119
Ca	0.5132579390	-1.9525762300	-3.1796460348

O	-0.4426068876	-2.8549358868	-1.2779361090
H	-0.8232536414	-3.7379625307	-1.2970297217
H	-0.1802851620	-2.6334830614	0.3125146523
O	-0.9195531614	-0.2044515727	-2.8722012389
H	-0.9334998121	0.5694636942	-3.4412641926
H	-1.4473553444	0.3504756783	-1.5454072134

12

17

i = 230, E = -1173.1700837698

O	1.5819322374	-2.3312657494	-0.6071670513
O	-1.3103129214	-1.8723275119	-2.8523434021
O	2.7618148051	-0.7988594072	-2.7052055484
C	0.8500487599	-1.7802218299	-1.6692090125
C	-0.6084007091	-2.1492180903	-1.6479304029
C	1.4797384812	-1.0830150695	-2.6287731428
H	2.9103038738	0.9058075827	-3.4166128894
H	-1.1442214797	-1.5992904930	-0.8668613287
H	-0.6863944638	-3.2149169342	-1.3902869807
H	0.8308329251	-0.7286144292	-3.4390648698
H	1.0714839757	-2.2319041993	0.2053784670
Ca	4.6513761049	-0.6492997988	-1.3361194423
O	3.1633269372	1.8499750102	-3.4655410561
H	3.5833995726	1.9407754386	-2.5683870562
H	-0.8483596734	-2.3304393713	-3.5638730117
O	4.3611336140	1.5869934391	-1.0572811392
H	4.0244110329	2.0932710069	-0.3158098565

13

17

i = 263, E = -1173.1528285086

O	1.5765242039	0.1668840223	-0.4439020078
O	-1.6383007095	0.0754430260	1.0628681485
O	-1.6863637432	1.4309145745	-1.3530148039
C	0.2427796720	0.3450473257	-0.5010809132
C	-0.6368582776	-0.5966463726	0.3013515123
C	-0.2938044900	1.3157373360	-1.2454039095
H	-1.9115276677	2.3603714008	-1.4664429757
H	-0.0071293850	-1.1525989102	0.9933943017
H	-1.1056944531	-1.3238067579	-0.3735042632
H	0.3007200834	2.0067632229	-1.8284123575
H	1.8524997168	-0.8048243228	-0.0600437032
Ca	2.3942150579	-3.9184243430	-1.0634643350
O	3.8446860966	-3.3644806144	-2.5843683534

H	4.5069422247	-3.4555993039	-3.2674726058
H	-2.0856732743	0.6636635605	0.4364766641
O	2.2549543994	-2.0709033150	0.3391283956
H	2.6308698327	-2.0151570468	1.2215533789

14

18

i =	105, E =	-1211.2428095361	
O	-0.1546519825	0.0507960628	1.2474656410
O	3.7575643036	-2.9482957834	-0.1169877826
O	-1.1705601862	-1.8974207610	-0.4940864766
O	-3.7373848357	-2.4510907552	-0.6345434721
C	-1.5114108688	-0.0684909620	0.9343359246
C	4.6832489871	-2.1939933423	-0.2761718821
C	-1.9575329336	-1.0083816030	0.0877640676
C	-3.4311974628	-1.1078066885	-0.2820899949
H	-2.1425170819	0.6581527482	1.4239595816
H	4.9107745303	-1.7602990642	-1.2614813139
H	5.3507432289	-1.9189132937	0.5532107434
H	-3.6630993431	-0.4413343183	-1.1237814680
H	-4.0563738039	-0.8042642297	0.5603589377
H	0.2550180408	-0.7300755174	0.8478309046
H	-2.8570322798	-2.8159458160	-0.8567783428
Ca	0.3160311877	-3.2377044599	-1.6057583142
O	-0.9333128988	-4.9662117079	-1.9245235251
H	-1.5101683304	-5.6453568054	-2.2663741105

15

14

i =	143, E =	-1096.7046356854	
O	0.5465968345	-1.2185160756	-1.8958655773
O	-0.5968185760	1.3255940305	0.4349653603
O	2.6457284485	-1.1371707351	-0.1723231277
C	0.6062886156	-0.1982334675	-1.0036207895
C	-0.7063693242	0.0693520001	-0.2362719882
C	1.7420219323	-0.3365504268	-0.0182945120
H	0.8390311034	0.7966056213	-1.4632664664
H	-0.9091584519	-0.7369484649	0.4790971075
H	-1.5144290140	0.0867330592	-0.9725786682
H	1.7464098148	0.3438374862	0.8502268347
Ca	2.4553535808	-2.3529981919	-2.5148243562
H	-1.4224974892	1.4951966497	0.8996863722
O	2.5338043484	-4.3467252255	-1.6437637495
H	2.2699519336	-4.9447550996	-0.9457978010

16

14

i =	87, E =	-1096.7103825028	
O	0.2824764826	-1.5319617452	-1.5780461409
O	-0.5928696158	1.2802967009	0.3743599006
O	2.7851486951	-0.7352704830	-0.9475191760
C	0.3887939195	-0.5867814702	-0.8095797603
C	-0.8693668833	0.0367954489	-0.2452546060
C	1.7421984053	-0.0519912005	-0.4141646002
H	1.6960541453	1.0215657125	-0.6958696170
H	-1.2859857658	-0.6843478119	0.4681427222
H	-1.5837539418	0.1383748302	-1.0678546195
H	1.7132003094	-0.0178656430	0.6960492744
Ca	2.5580743311	-2.2817606811	-2.5815712287
H	-1.3861300249	1.5682595101	0.8357504673
O	2.4817658173	-4.2978866077	-1.7462223542
H	2.2647605746	-4.8476798731	-0.9942433520

17

17

i =	305, E =	-1173.1657779025	
O	0.1869268945	-1.9813870618	-0.2419960829
O	-1.6636963955	-0.6274528696	1.0831501632
O	2.2084162484	-1.0055397114	-1.8083382500
C	0.3088125007	-0.7724512123	-0.3129522281
C	-0.6758517703	0.1166097710	0.4103752037
C	1.4098699754	-0.1120677454	-1.1084494700
H	0.9236180532	0.5879581031	-1.7975851544
H	-1.1313434339	0.7840700969	-0.3276199009
H	-0.1141281522	0.7420549822	1.1113234692
H	1.9770526594	0.5079986962	-0.3981238909
Ca	1.6433755982	-3.7937168731	-1.3489593781
H	-1.4521683468	-1.5612758320	0.9309440887
O	1.0623504963	-5.5910594286	-2.3777185402
H	0.9956681011	-6.2868480217	-3.0278194678
O	3.3023032055	-2.5591044123	-0.2815829924
H	4.2549746897	-2.6618813763	-0.3355512147
H	2.8066133056	-1.5635669157	-1.1157079291

18

21

i =	159, E =	-1287.7302960980	
O	-0.7214124965	1.7321792434	-1.7824702176

O	1.2509104937	-1.3533764906	-1.5044869392
O	-1.6363700261	-1.5978613404	-1.1007298133
O	-3.8577734151	-0.7998215801	-2.2629346605
C	-0.4062037485	0.4368910487	-1.2977753453
C	0.7842867656	-0.1965025845	-2.0735208389
C	-1.6212931330	-0.4281730406	-1.4508550303
C	-2.8477222010	0.1627323514	-2.0936166271
H	-0.1499196716	0.4574585162	-0.2335264173
H	0.4460162318	-0.3303120367	-3.1179799804
H	1.5450563863	0.6046199089	-2.1056628453
H	-2.5543955320	0.6025461637	-3.0504258193
H	-3.1835534563	0.9904825758	-1.4600250419
H	-0.0379897553	2.3385494465	-1.4763941264
H	-3.5191589225	-1.6172330394	-1.8654435859
Ca	0.2690006200	-3.3640773362	-0.7588829196
O	-0.1422393815	-5.4498110162	-0.4454028332
H	-0.1326358278	-6.4039144114	-0.4214046804
O	0.9991029715	-1.4616578936	1.0163850323
H	1.8135122534	-1.5233118222	1.5264101699
H	1.2623136143	-1.2236310878	0.0672483768

19

21

i = 341, E = -1287.7364195301

O	-0.8669938951	1.6466947589	-1.9762177203
O	1.0648143914	-0.4006154558	-2.4967652385
O	-1.3486077515	-1.3171501943	-0.1420921244
O	-2.8692148846	-1.9352137601	-2.3123237421
C	-0.4708120561	0.7105323319	-0.9822735998
C	0.9589542214	0.2305368258	-1.2302068829
C	-1.3866888007	-0.5025208937	-1.0439062977
C	-2.2704016982	-0.6835823953	-2.2504936057
H	-0.5419662541	1.1366863241	0.0227968769
H	1.6409645540	1.0824895147	-1.1540802986
H	1.2298692504	-0.5082912605	-0.4767257021
H	-1.6450971125	-0.5053661696	-3.1305578844
H	-2.9948157006	0.1442106204	-2.2350675511
H	-0.4530472716	2.4950703119	-1.7784189205
H	-3.6003615437	-2.0048023910	-1.5524897408
Ca	-2.4706577767	-3.6178948254	0.0882003896
O	-4.3463340067	-2.3036169278	-0.3737379033
H	-5.2458417460	-2.5326068145	-0.6157242329
O	-0.6342484948	-4.4852766503	-0.7269216633
H	0.1386133104	-4.1814198965	-1.2027542967

H	0.8086575039	0.2483282691	-3.1644043165

20

21

i =	112, E =	-1287.7409434524	
O	0.6110584619	1.7347922920	-1.1267630256
O	1.3210601939	-1.7053571547	-2.0401205050
O	-0.7754726410	-1.0714703102	0.5160064297
O	-1.6366475270	0.8390224858	-2.4807719246
C	0.6773736193	0.3270088452	-0.8108996968
C	1.4015276875	-0.3062096031	-2.0022050717
C	-0.6922769182	-0.2930313235	-0.5387786980
C	-1.7215884597	-0.0160861604	-1.3710806908
H	1.2670032138	0.1823717861	0.0981840171
H	0.9365337666	0.0826544706	-2.9140001699
H	2.4420034667	0.0524430322	-1.9939798993
H	-2.7175014336	-0.4077912626	-1.2064666532
H	-1.0727216793	-2.3365257847	-1.6351730661
H	0.2932920080	2.1998654197	-0.3438247594
H	-0.8964805028	1.4393839654	-2.2974163374
Ca	-0.4456851812	-3.2885294013	1.0099183946
O	-1.3347467957	-3.2529583551	-1.4236012316
H	-2.2814227407	-3.2888246598	-1.6011037713
O	1.6430389028	-3.0024669670	0.1676813201
H	2.3512324883	-3.6357413934	0.0270027276
H	1.5035076174	-2.1357466123	-1.1330518983

21

21

i =	127, E =	-1287.7369633898	
O	-1.0471094468	-0.3127876262	1.4190560362
O	1.3274985255	-0.7143651286	-1.3699078837
O	-2.1107686851	-0.2628939450	-1.2026214414
O	-1.2513683658	-3.7418713611	-0.4758775590
C	-0.4479353444	-1.0155576887	0.3178289147
C	0.6699082122	-0.1449889974	-0.2705438302
C	-1.4830084562	-1.3907056458	-0.6909131400
C	-1.7974233254	-2.6368794837	-1.0402210574
H	-0.0271143551	-1.9358372277	0.7193251250
H	0.2220057676	0.8002498718	-0.5953199530
H	1.3590902631	0.0876996768	0.5535729686
H	-2.5677633817	-2.7941200503	-1.8002355697
H	-2.8901041321	-0.5196201874	-1.7096958747
H	-1.6259021113	0.3574941809	1.0326761355

H	-0.6257628493	-4.2522078516	-1.1776084762
Ca	2.4453626771	-4.5050959748	-2.4803706872
O	0.2232100837	-4.9483483766	-2.0640009187
H	-0.3107030351	-5.6058586828	-2.5168688371
O	2.6833740961	-2.8783837686	-0.9012855898
H	2.9028069468	-2.9824707979	0.0268160424
H	1.8245077289	-1.5593081075	-1.0968400298

22

21

i = 236, E = -1287.7368257902

O	-1.1464043950	-0.1603729188	1.3898300863
O	1.3985328988	-0.7498223685	-1.2081104309
O	-2.0963911438	-0.2131008948	-1.3172853132
O	-1.4401228296	-3.7036230930	-0.4150052321
C	-0.5245878044	-0.9416373638	0.3411781516
C	0.6513438114	-0.1202217285	-0.1988234595
C	-1.5191144323	-1.3374877235	-0.6884711223
C	-1.8823770816	-2.5997952365	-0.9722518710
H	-0.1506038521	-1.8557187141	0.8016921863
H	0.2465547988	0.8082624297	-0.6157650858
H	1.2732796838	0.1576158353	0.6650512685
H	-2.6485396910	-2.7000687298	-1.7650795963
H	-2.9577923554	-0.4679067783	-1.6661594156
H	-1.7498759150	0.4428170539	0.9367844234
H	-0.4354468481	-4.4002120601	-1.2503971523
Ca	2.5609192979	-4.5594127160	-2.3977834830
O	0.2092538519	-4.9518478475	-1.8499949545
H	-0.3634670725	-5.4849793960	-2.4094826270
O	2.5764941292	-3.0374512027	-0.7050878858
H	3.1273932288	-2.9836273357	0.0789372186
H	1.7986396365	-1.6265291249	-0.8968611602

23

21

i = 267, E = -1287.7390841050

O	0.8002835998	1.5900277172	-2.2319712570
O	2.1083786477	-1.7011128022	-1.7388358273
O	-0.7443306879	-1.6695815104	-2.9081484918
O	-1.9338902218	1.3803834900	-1.6398631104
C	0.6287783103	0.1889638351	-2.0176073814
C	1.9041466788	-0.5137185434	-2.4657885251
C	-0.6089523652	-0.2875795927	-2.7941825135
C	-1.8716712118	0.3113600756	-2.2064832371

H	0.4879309267	-0.0225595868	-0.9510147523
H	1.8526504826	-0.7099802377	-3.5432656588
H	2.7254527595	0.1954145720	-2.2966865517
H	-0.5122918430	0.1465500056	-3.8019220921
H	-2.7707050022	-0.3224078891	-2.3203829512
H	-0.0217765140	2.0092534354	-1.9382719808
Ca	0.7118453160	-4.2151666096	-2.1577493107
O	2.7389094400	-3.6849929225	-3.1155577502
H	3.6045144377	-4.0816309888	-2.9974188083
H	2.5490676848	-2.4072354668	-2.3395330024
O	-0.7443609697	-2.9859991147	-0.8175184920
H	-0.8261517722	-2.1221749764	-1.9536855165
H	-1.6137111935	-3.2973517402	-0.5508641641

24

18

i = 80, E = -1211.2722973269

O	-1.0019850404	1.7462141484	-2.0746936805
O	1.7087169503	-0.7372915903	-1.8518769662
O	-0.8554562281	-1.7072186005	-1.3581956064
O	-3.4620766439	-1.5623155379	-1.9822871124
C	-0.3472678609	0.5886883800	-1.5884543475
C	0.9131435010	0.2908538505	-2.4159841932
C	-1.2947614363	-0.5959658548	-1.6365455159
C	-2.7322780847	-0.4236559579	-2.0496757953
H	-0.0513037451	0.7104146222	-0.5388161350
H	0.6093301183	0.0474642126	-3.4412336122
H	1.5215150820	1.1946358144	-2.4478931044
H	-2.6516233856	0.0265128028	-3.0651171985
H	-3.1074599058	0.4233212578	-1.4384863186
H	-0.5343785535	2.5210437003	-1.7442663425
Ca	-2.6098523899	-3.6609132888	-1.6805437232
O	-2.8384562517	-5.6169790858	-0.7884587909
H	-2.9733963613	-6.4932742511	-0.4343723040
H	1.1040283165	-1.4648565474	-1.6390320841

25

18

i = 82, E = -1211.2772779007

O	-1.0124996182	1.7856962982	-2.2055994233
O	1.4541018479	-0.9030871628	-1.7885553063
O	-1.0041563147	-1.8317901285	-1.9206970608
O	-3.5261212849	-1.2301187761	-1.1639790862
C	-0.4944563144	0.5463387855	-1.7280582402

C	0.8858937783	0.2793551485	-2.3198002641
C	-1.4655945431	-0.5687619181	-2.1690402134
C	-2.8390488770	-0.3248876063	-1.5992092330
H	-0.4146371174	0.5541005028	-0.6339715605
H	0.7790146990	0.2207982281	-3.4126565145
H	1.5499319677	1.1186754247	-2.0990781283
H	-1.6251739041	-0.3616994933	-3.2547004417
H	-3.2259740163	0.7063384089	-1.6257948719
H	-0.5728793226	2.5015986575	-1.7327475510
Ca	-2.5090735458	-3.6109011580	-1.6215673562
O	-2.8700029448	-5.5690957155	-0.7862843815
H	-2.8097932917	-6.4658499606	-0.4614790202
H	0.6787294886	-1.5209616859	-1.7787063648

26

18

i = 269, E = -1211.2647408778

O	2.5542953128	1.0192022036	0.1532439011
O	1.7916345363	-1.3287489281	-2.5201352896
O	3.1118216967	-2.5476122981	-0.4338572102
O	0.5985951132	1.9295735900	-1.7972302214
C	2.2847174215	-0.1944491578	-0.3921093843
C	1.9599842534	-0.0470093572	-1.9156771188
C	3.4579969172	-1.1796656281	-0.2307549733
C	0.6939682261	0.7457976608	-2.0388069597
H	1.4058680124	-0.7034635143	0.0639144384
H	2.7945061651	0.4983010282	-2.3655851391
H	3.8112643354	-1.0848130923	0.7965444850
H	4.2755907964	-0.8805997064	-0.9010811616
H	-0.1965975974	0.1574413133	-2.3294797072
H	1.9931206676	-1.2643728954	-3.4600281787
H	2.6731422162	-2.5699704705	-1.2969771411
Ca	2.1048882344	3.2364025473	0.0026287254
O	2.2660455725	5.3189395882	0.5642013951
H	2.4886627015	6.1362790936	1.0042602715

4+5

18

i = 141, E = -1211.2458566809

O	2.8794657323	1.0540014637	0.4231324015
O	1.6631991028	-1.3786527493	-2.8520094666
O	2.8041853834	-2.3816808249	-0.5220543212
O	0.9106889050	1.9510016626	-1.6247241084
C	2.5137374485	-0.0880287686	0.2339933738

C	1.8781609241	-0.0222948522	-2.5145662489
C	3.4673043744	-1.2129451108	-0.0987898578
C	0.8562071682	0.7289753320	-2.0462976683
H	1.4649472343	-0.3916150239	0.3871669486
H	2.8948814448	0.3374068058	-2.6268119452
H	4.0065583523	-1.4458909991	0.8251187573
H	4.2015661097	-0.8527704302	-0.8251763077
H	-0.1180842861	0.2105244842	-2.0078163581
H	1.9340974877	-1.5211654356	-3.7653279075
H	2.4262586648	-2.1999663600	-1.4043516363
Ca	1.9413432222	3.4227103369	-0.1463378125
O	2.3510106358	5.3856237035	0.6259902382
H	2.3644443321	6.3060476822	0.8774277128

TS1

9

Step = 0, E = 0.00000000

O	1.6266922215	5.0264390326	15.9158396638
C	2.2885848493	5.4751850626	16.8682271064
H	2.1705473729	6.5942992955	16.9845558608
H	3.0104319409	4.2990507007	17.4828559939
O	3.2890255039	3.2009752852	17.5740000899
H	4.2458545602	3.1555441610	17.6673386905
Ca	1.9481640307	2.5383067970	15.6492106232
O	0.2251364261	1.9733215149	16.9240929035
H	0.2401426723	2.1458813753	17.8681142958

TS2

10

Step = 0, E = 0.00000000

Ca	0.3534145829	1.6924725467	-2.6108886613
O	-0.7234884013	-0.1195003719	-2.0115915608
H	-0.8680598939	-0.8504294223	-1.4132617374
H	3.7813228448	1.6233525557	0.0148390583
C	3.2116872282	1.0978139808	-0.8910290284
O	3.9389278030	1.0661856602	-1.8866140982
C	3.1077223356	-1.1170316717	0.8169853045
H	3.3385186008	-0.1422797977	1.2726192327
H	3.7645692244	-1.4291049690	-0.0094895285
O	2.2317751144	-1.8359701232	1.2399098717

TS3

13

Step = 0, E = 0.00000000

O	0.2335430301	0.8213458854	-0.1974477820
O	0.3194431863	0.1240599452	2.4521521550
C	-0.2607316383	-0.4091011776	0.1937887631
C	-0.1624282642	-0.6652045696	1.6661135747
H	0.2396651297	-1.2650440665	-0.2918418311
H	-1.3247435867	-0.5158778522	-0.0609261184
H	-0.5588263013	-1.6292710625	2.0228280666
H	1.3455182978	0.8344466314	-0.0456524394
O	2.5384960028	1.0465014566	0.4345883209
H	3.1634432983	1.2752129592	-0.2574030321
Ca	1.2104828686	2.4292285528	1.8191051441
O	0.7662878189	4.3752089162	2.5909201280
H	0.6789026849	5.2647712835	2.9241981140

TS4

13

Step = 0, E = 0.00000000

O	1.7365146947	-0.1686200799	0.1514853991
O	-1.8316808925	-0.0712194640	0.0491939929
C	0.4262658669	-0.6172930301	0.4752779507
C	-0.6246553592	0.1847175358	-0.0685382390
H	0.0253348648	-0.4957434949	1.7291549123
H	0.3003250935	-1.6914921179	0.3145566903
H	-0.3161604185	1.1508202203	-0.5109904063
H	2.1330988135	0.1823756330	0.9537906352
Ca	-2.9517826452	-0.5516256581	2.2587538150
O	-5.0394006826	-0.7889652744	2.6852328100
H	-5.9535826543	-0.9481075063	2.9062021949
O	-0.6882122734	-0.4174079710	2.8348062966
H	-0.1919360603	-0.8424167813	3.5397486621

TS5

14

Step = 0, E = 0.00000000

Ca	0.614948	1.910242	-0.521654
O	-1.489500	2.215617	-0.724787
H	-2.435860	2.348485	-0.751458
C	3.219867	1.151419	0.987078
H	3.810553	0.595568	1.770516
C	3.787687	2.312952	0.643493

H	3.373624	3.033184	-0.072000
O	2.145949	0.583335	0.478984
O	4.962078	2.715912	1.322806
H	5.629312	2.980732	0.675158
C	3.534178	1.628616	-2.163568
H	4.318956	2.153638	-2.735786
H	3.810432	0.690726	-1.664236
O	2.401476	1.980646	-2.231511

TS6

17

Step = 0, E = 0.00000000

O	-0.1399748512	1.6752794405	0.1644102212
O	1.9598832781	-1.2484442338	-0.4092295379
O	-1.6899287180	-0.3888564426	0.8066457183
C	0.2210033468	0.4148306680	-0.3524767894
C	1.6447273939	0.0161906017	0.0740227721
C	-0.7468960364	-0.6446660425	0.0891982524
H	0.1898700631	0.4316531558	-1.4500039668
H	1.6748206181	0.0680055773	1.1750698826
H	2.3243167140	0.7992446853	-0.2938602800
H	-0.5431024120	-1.6638354890	-0.2613829076
H	-0.9232963938	1.5333002273	0.7193878566
Ca	2.5320709038	-2.7968718943	-2.5944340747
O	4.1625162902	-1.7766667355	-1.1348133586
H	4.6987718455	-2.2325079214	-0.4818631107
H	3.1113915470	-1.4153205091	-0.6188835599
O	1.2763963237	-4.4236321358	-3.2468427657
H	0.8951249152	-5.1637225036	-3.7144945847

TS7

22

Step = 0, E = 0.00000000

O	0.9641406245	1.0297322565	0.6935815526
O	0.6559886827	-1.6614709427	0.2693375107
O	-2.1201806502	-2.5117928981	1.4805404047
O	-1.6148256941	-0.6498552175	-0.7770411192
O	1.2151005268	1.9009590559	3.4218728920
C	0.3996874121	-1.2143508443	1.4071445737
C	1.0072976211	0.1249606849	1.7860448021
C	-1.9191313383	-1.1725423355	1.5243796208
C	-1.7802389666	-0.3226650172	0.4515051991
C	0.4271940417	0.7841000778	3.0198864349
H	0.1758288695	-1.8953047795	2.2372724701

H	2.0609781406	-0.1066990095	2.0106368186
H	-2.0949024206	-0.7306317836	2.5025223322
H	-1.7418322453	0.7405793416	0.7294666138
H	0.4409126979	0.0755366770	3.8489085852
H	-0.6062818365	1.0843628848	2.8306982710
H	-1.9712717290	-2.9365077033	0.5378874166
H	1.2606469666	2.5037674074	2.6695849698
Ca	-0.3339472225	-2.3520067356	-1.8398197149
O	-1.7653535070	-3.7232612326	-0.7160240114
H	-1.6271037350	-4.6333561995	-0.4432637995
H	0.6932937616	0.5054463129	-0.0761218230

TS8

22

Step = 0, E = 0.00000000

O	0.6063622115	0.6307222583	0.2027673460
O	0.1679720901	-1.6408237424	-0.1462832592
O	-2.0358239022	-2.6301683035	1.0243932251
O	-2.9450781539	-0.3700992703	-0.2026698840
O	-0.6649756954	1.7051722886	2.5781418368
C	-0.0228299111	-1.3696031313	1.2071941874
C	0.7376421733	-0.0302430465	1.4407832207
C	-1.5478416168	-1.3804361289	1.4713460373
C	-2.2140295718	-0.2322739862	0.7547453000
C	0.3908067713	0.7852020194	2.6947539633
H	0.4015781558	-2.1460177387	1.8627687147
H	1.7891284916	-0.3350002292	1.5804057902
H	-1.7568503669	-1.2822055914	2.5420687417
H	-1.9704854853	0.7577055384	1.1649902375
H	1.3020434469	1.3023412518	3.0254130752
H	0.1425107953	0.0658720994	3.4871517440
H	-1.5586838284	-2.7489441113	0.1836718805
H	-0.3179351605	2.4647716326	1.9879663955
Ca	0.1566649009	2.6455154351	-1.0394076112
O	0.2255144054	3.5909511678	1.0550352159
H	0.9847969461	3.9713770124	1.5055101559
H	0.4055133042	-0.3868154241	-0.3287463134

TS9

22

Step = 0, E = 0.00000000

O	0.6193506504	0.1534336379	0.9250226580
O	-0.0236655919	-2.5933732067	1.6793157114
O	-2.4748414047	-2.0667795469	0.5554726101

O	-1.5076036090	-0.0401153646	-0.9753289153
O	0.7163464518	1.9408764782	3.1613194972
C	-0.6761026256	-1.4285506649	2.1366932737
C	0.3352919875	-0.2795070927	2.2095418892
C	-1.8623625693	-0.9917698657	1.2203129263
C	-1.4917973351	0.1263273122	0.2327500149
C	-0.1808663672	0.8375213676	3.1217438968
H	-1.0700512057	-1.6590164158	3.1353423620
H	1.2349650065	-0.6877667625	2.7037119723
H	-2.6207102395	-0.5459858729	1.8797139160
H	-1.4933687385	1.1392193530	0.6561039550
H	-0.2801195723	0.4625988854	4.1436204394
H	-1.1744926578	1.1816202459	2.7985999270
H	-1.8291786637	-2.3676217313	-0.1373053732
H	0.9153631831	2.1338122034	2.2354460086
Ca	1.4020147175	-1.2485518374	-0.8365549247
O	-0.3297486940	-2.8427933728	-0.8860506361
H	-0.3152641046	-3.7728815722	-1.1261840482
H	-0.2221586179	-2.7706961772	0.7097128396

TS10

25

Step = 0, E = 0.00000000

O	0.0232516673	0.5836211276	0.5797696642
O	-0.0912382531	-2.5116802431	1.2451570210
O	-2.5265197101	-1.6463524212	0.2424263196
O	-1.8405460706	0.8614290317	-0.7740077556
O	0.9848648396	1.9410211982	2.7992470011
C	-0.6099892423	-1.3349634982	1.8155290212
C	0.3630485450	-0.1573200217	1.7451223064
C	-1.8878932437	-0.7608630835	1.1204857422
C	-1.4401743757	0.5983683907	0.4772764822
C	0.2781505022	0.7232424782	2.9877166677
H	-0.8230683219	-1.5642757146	2.8655799568
H	1.3874555596	-0.5124577040	1.6211521356
H	-2.6184273655	-0.5235500245	1.8985556832
H	-1.7808110399	1.4098241046	1.1491914153
H	0.7301397241	0.1986731635	3.8306261907
H	-0.7709320085	0.9217462468	3.2433724059
H	-1.8811350722	-1.9616326291	-0.4455299924
H	0.7795695794	2.2202708686	1.8958146671
Ca	0.4599367580	-1.9690457368	-3.1581975012
O	-0.6711646025	-2.8111136262	-1.3076367015
H	-1.0662265553	-3.6880197383	-1.3033997758

H	-0.2733973366	-2.5708547201	0.2598953124
O	-1.0059270016	-0.1874753506	-2.7826644216
H	-1.1135908692	0.5387550366	-3.4026571839
H	-1.3942709220	0.2541412906	-1.6984463080

TS11

17

Step = 0, E = 0.00000000

O	0.1588721110	1.6653790401	0.2991067183
O	1.9761225773	-1.5313122323	0.1961463007
O	-1.8983484096	-0.0065765939	0.7022873364
C	0.3396172730	0.2722146283	0.0400590185
C	1.7129787613	-0.1884064899	0.5178820012
C	-0.7895173972	-0.4982335263	0.4184205949
H	0.4263483501	0.1012680268	-1.3332884924
H	1.7914136138	-0.0497405741	1.6064890829
H	2.4654461512	0.4706134994	0.0649014174
H	-0.6619297465	-1.5913684337	0.3911027920
H	-0.7500836131	1.7285621816	0.6340006155
Ca	2.7405452089	0.0020500848	-3.6204726150
O	3.4001244324	-1.4028099858	-1.9534433153
H	4.2827081401	-1.6436848499	-1.6627289785
H	2.5050885734	-1.5177596004	-0.6735235161
O	0.6327745816	0.1019861827	-2.5688671199
H	-0.1741606077	-0.2681813573	-2.9390718406

TS12

17

Step = 0, E = 0.00000000

O	1.5119444260	-2.0101204205	-0.7729122876
O	-1.5704375418	-1.9998478408	-2.7896297410
O	2.4178890354	-0.6136095877	-2.9615873183
C	0.6504341011	-1.6556349194	-1.7961145358
C	-0.7539141439	-2.1731491919	-1.6453098907
C	1.1090373165	-0.9416528726	-2.8244645956
H	2.6451352413	0.3936793526	-3.2687269881
H	-1.2571053024	-1.6507887338	-0.8281333157
H	-0.7018613332	-3.2330818171	-1.3628638795
H	0.4175101293	-0.6627320813	-3.6142921119
H	1.0224987435	-2.4549557383	-0.0718364984
Ca	4.4098731920	0.6882296922	-1.3904254716
O	3.4248870382	1.5444643163	-3.3387495136
H	2.9653389505	2.3782666911	-3.4548514812
H	-1.2234165038	-2.5689754269	-3.4868344783

O	3.9390647517	2.1880384916	0.0786540259
H	4.0660450805	2.8364032586	0.7685857550

TS13

17

Step = 0, E = 0.00000000

O	1.5751403904	0.1666493072	-0.4095132632
O	-1.6556625168	0.0891664276	1.0545938148
O	-1.6813529320	1.4126194116	-1.3696992837
C	0.2493799269	0.3429528141	-0.4852534993
C	-0.6481500988	-0.5917497028	0.3073370888
C	-0.2871345793	1.3058102130	-1.2439404761
H	-1.9127658676	2.3458965341	-1.4311458530
H	-0.0348054855	-1.1511508353	1.0144047273
H	-1.1129579864	-1.3164379361	-0.3711219025
H	0.3104617070	1.9936367384	-1.8289237184
H	1.8821781491	-0.8466938985	-0.0240435624
Ca	2.4653011736	-3.8680101593	-1.0967722555
O	3.8922911393	-3.3093967639	-2.6476697845
H	4.5448317936	-3.4863893297	-3.3243258781
H	-2.0867683109	0.6768802942	0.4169456141
O	2.2972940274	-2.0284944673	0.3363011681
H	2.6364049906	-1.9681050604	1.2330799869

TS14

17

Step = 0, E = 0.00000000

O	1.4176643823	0.3082727442	0.1822430890
O	1.5885642786	-2.4811470653	0.2199020146
O	2.1804848340	-0.6023734810	3.6526860644
C	1.3382502946	-1.8721684545	1.2342383690
C	1.2059656439	-0.3633569174	1.3404896750
C	2.1673285223	0.1484514426	2.4405291160
H	1.1642892380	-2.4234051817	2.1780577302
H	0.1730690752	-0.2479850189	1.7549692100
H	1.9199624440	1.1961473868	2.6319194728
H	3.1805394052	0.1128097788	2.0363625175
H	1.3436349211	-0.4634284278	4.1078001936
Ca	1.7164192445	2.1617430144	-2.6269231486
O	2.6873266227	3.5925631176	-1.2242117768
H	2.4452785718	3.6564470297	-0.2980630742
H	0.4852254658	1.1439242121	-0.3608858499
O	-0.0914503551	1.8370134248	-0.9688267858
H	-0.9075525890	1.3834923956	-1.1992868167

TS15

14

Step = 0, E = 0.00000000

O	0.3558620739	-1.3864629071	-1.7073404982
O	-0.5695993787	1.3411958198	0.4054769208
O	2.7790325594	-0.9206877969	-0.4764188900
C	0.4583006726	-0.5261791833	-0.7675509476
C	-0.8175957644	0.0825342345	-0.2052140338
C	1.7245481309	-0.2980922947	-0.1222748091
H	1.1986005035	0.6258242918	-1.0436353541
H	-1.2274022262	-0.6279247299	0.5213037476
H	-1.5248781512	0.1648173764	-1.0344002223
H	1.7158938749	0.3738718828	0.7432486188
Ca	2.4471314238	-2.3849967202	-2.4084553961
H	-1.3774551721	1.6233778111	0.8490100380
O	2.4148962214	-4.3250493462	-1.4752054692
H	2.2845604167	-5.0942471090	-0.9244864921

TS16

17

Step = 0, E = 0.00000000

O	0.2236469797	-2.0131308914	-0.1643311284
O	-1.6567727611	-0.6560869566	1.0873203234
O	2.2146082453	-1.0881630249	-1.7747758999
C	0.3247496862	-0.8049981862	-0.2876064099
C	-0.6802473107	0.0890209447	0.3990399534
C	1.4199390011	-0.1716804845	-1.1114728384
H	0.9266308245	0.5117137998	-1.8154088879
H	-1.1464232641	0.7212392742	-0.3625910291
H	-0.1361046753	0.7488099694	1.0824281589
H	1.9837896425	0.4754186875	-0.4192520452
Ca	1.6552010698	-3.7650984741	-1.3534098477
H	-1.4068897792	-1.5874188525	0.9856883505
O	1.0787582791	-5.5350431812	-2.4137821320
H	0.9360100426	-6.2922428200	-2.9763618152
O	3.3711176535	-2.5319758190	-0.2597793823
H	4.3209365948	-2.6017656710	-0.3823625114
H	2.8780497713	-1.6525983142	-1.0243428590

TS17

17

Step = 0, E = 0.00000000

O	1.1316653941	-0.9068943577	0.5963218232

O	-1.2842509243	-1.8876787935	0.7348393879
O	-0.3301060475	1.3085819449	-1.7963817153
C	0.3186609323	-0.3131566006	-0.1388572355
C	-1.1330548076	-0.7371954937	-0.0684634456
C	0.7317043510	0.6452552975	-1.1187479313
H	-0.2788928902	1.0699088558	-2.7265062982
H	-1.5045833854	-0.9152869434	-1.0783424592
H	-1.7018242814	0.1075064183	0.3372701899
H	1.4854450740	1.3410651026	-0.7442018259
H	1.4866595209	-0.2073236145	-1.8043725086
Ca	3.4707022574	-1.6438476186	-0.1261551325
O	4.3838186140	-3.5322371908	-0.5982255002
H	4.5003108879	-4.4404452179	-0.8693356819
H	-0.4043468324	-2.0350837025	1.1217308620
O	2.3540761277	-1.1306862640	-2.1113971628
H	2.6890160096	-0.9684818220	-2.9971753660

TS18

18

Step = 0, E = 0.00000000

O	0.587437	-2.285488	1.163996
O	1.762949	-0.137755	-0.629506
O	-1.394212	-1.896455	-0.714627
O	-3.299352	-0.139802	-0.606018
C	-0.253426	-1.146844	1.192129
C	1.677173	0.033796	0.575359
C	-1.234552	-1.059237	0.234523
C	-2.205735	0.125290	0.258559
H	-0.464294	-0.718886	2.165537
H	1.132733	0.860588	1.055305
H	2.310029	-0.558171	1.260765
H	-1.688703	1.038667	-0.061791
H	-2.566665	0.283485	1.277454
H	0.103345	-2.919539	0.622518
H	-3.046723	-0.994203	-1.010163
Ca	0.962479	-4.979966	-1.922186
O	-1.091286	-5.737296	-2.183505
H	-1.883513	-6.013845	-2.642019

TS19

21

Step = 0, E = 0.00000000

O	-0.7247298450	1.7464880449	-1.7603152631
O	1.3239436869	-1.2907933842	-1.4636843792

O	-1.6026270908	-1.6113238071	-1.1882278368
O	-3.8654661636	-0.7802010338	-2.2509114583
C	-0.4026582382	0.4484579815	-1.2937965670
C	0.8162593588	-0.1342521711	-2.0472729589
C	-1.6089639218	-0.4276114672	-1.4833705041
C	-2.8535727500	0.1783129437	-2.0745489133
H	-0.1718709786	0.4419631289	-0.2223270152
H	0.5219424653	-0.3250036616	-3.0870698135
H	1.5587779777	0.6773155553	-2.0720338010
H	-2.5820706616	0.6478683116	-3.0240562018
H	-3.1759582616	0.9860746937	-1.4093727007
H	-0.0510955324	2.3577191306	-1.4413919433
H	-3.5168055818	-1.6136707339	-1.8987487342
Ca	0.1541505824	-3.3627628116	-0.4550523725
O	-0.1719574838	-5.4710474312	-0.4136292721
H	-0.1667927553	-6.4242534220	-0.3800861819
O	0.9419518485	-1.5145777923	0.8753383342
H	1.6966019565	-1.4836797149	1.4693641182
H	1.2717669727	-1.2359034557	-0.3140995062

TS20

21

Step = 0, E = 0.00000000

O	0.5085713648	1.7761936814	-1.2568676421
O	1.6174225646	-1.5924519945	-2.0817007343
O	-0.5254672403	-0.9773268756	0.5226965766
O	-1.5601384244	0.4923319354	-2.5923826484
C	0.7829254809	0.4125980789	-0.9031936983
C	1.5207932496	-0.1960519079	-2.0974633329
C	-0.5059308878	-0.3205141211	-0.5370919442
C	-1.6256018722	-0.3172410226	-1.4258443032
H	1.4153860562	0.3737624799	-0.0142447220
H	0.9745524417	0.1008269152	-2.9970020258
H	2.5073480646	0.2886294976	-2.1393225340
H	-2.5801377232	-0.2285846132	-0.8971072732
H	-1.5902289970	-1.6445418720	-1.6419185077
H	0.4213283168	2.2941665151	-0.4490770887
H	-1.1011571381	1.3079927013	-2.3461413767
Ca	-0.4799978933	-3.4640140939	0.4974666720
O	-1.5393412761	-2.9346760600	-1.5316148336
H	-2.3497412638	-3.2703356085	-1.9251305859
O	1.6935039688	-2.9227451703	0.1300155551
H	2.4348742410	-3.5267216591	0.0377858952
H	1.6899951917	-2.0052592507	-1.1534934994

TS21

21

Step = 0, E = 0.00000000

O	-0.5412444563	1.2114727739	-0.7589916069
O	1.3756727303	-1.6931671411	-1.8017703603
O	-0.9685219909	-2.0613758041	0.5824683185
O	-2.3988617778	-0.1942661153	-2.2238867385
C	-0.0292560961	-0.1177171531	-0.5143341491
C	0.9362914101	-0.3711194936	-1.6763354844
C	-1.1270342924	-1.1603677421	-0.4062113930
C	-2.1943781917	-1.1276582159	-1.2120755207
H	0.5149611138	-0.1281683548	0.4333804968
H	0.3985648631	-0.1130666017	-2.5946448480
H	1.7683506860	0.3436519699	-1.5826112758
H	-2.9951462942	-1.8516286259	-1.1327591689
H	-0.6496485726	-3.1154309082	0.2585052833
H	-1.0593789156	1.4755703384	0.0102878141
H	-1.8305952133	0.5646383479	-2.0086881966
Ca	1.8537776392	-5.1454893913	-0.0022555835
O	-0.3248964529	-4.3330769096	0.0317076203
H	-1.1333222195	-4.8464787406	0.0979316472
O	2.5490759781	-2.9800416761	0.1288335910
H	3.4312187646	-2.6361909992	0.2849706485
H	1.8074450188	-2.0681213407	-0.9633580609

TS22

21

Step = 0, E = 0.00000000

O	-1.1734743222	-0.1602565358	1.3830753907
O	1.3843366955	-0.7641086459	-1.1972879339
O	-2.1209510695	-0.2292245694	-1.2657469078
O	-1.3345068300	-3.7024616993	-0.4176549579
C	-0.5282480544	-0.9384764392	0.3563083090
C	0.6435223003	-0.1191411423	-0.1965161625
C	-1.5171979027	-1.3457099048	-0.6837260585
C	-1.8343087267	-2.6018899387	-1.0025211205
H	-0.1494323689	-1.8437920730	0.8265416940
H	0.2356390658	0.8035853699	-0.6245592734
H	1.2634115615	0.1683724363	0.6642843220
H	-2.5856323568	-2.7499122755	-1.7878478856
H	-2.9396693293	-0.4954279690	-1.6999286856
H	-1.7490026671	0.4636745592	0.9209062106
H	-0.6123400995	-4.2681710308	-1.1210010679

Ca	2.4382145308	-4.5187097736	-2.3380567053
O	0.1758969912	-4.9242526254	-1.8672592004
H	-0.3804167596	-5.4681579420	-2.4310064334
O	2.5679096507	-3.0151483018	-0.6372207158
H	3.1586339916	-2.9509060029	0.1159213122
H	1.8007483296	-1.6301299004	-0.8647302357

TS23

21

Step = 0, E = 0.00000000

O	-1.6530600313	-0.4994527714	-0.0299699931
O	1.6949718479	-0.9164285228	1.3145744375
O	0.6022755482	-2.3136569818	-1.5180099853
O	-2.5487187579	-3.2218288093	-0.1591372794
C	-0.5620951016	-1.3014413487	0.4693402841
C	0.6164209759	-0.3465166569	0.6220060831
C	-0.2974921212	-2.5090604774	-0.4140588452
C	-1.4863464657	-3.2315715830	-0.7854418268
H	-0.8294101463	-1.6625986211	1.4696625261
H	0.9219788652	0.0111837926	-0.3678021577
H	0.2357983830	0.5201505126	1.1752584730
H	-1.3856323763	-3.8819338159	-1.6771478866
H	0.3145429857	-1.5666344878	-2.0538233184
H	-2.4132067832	-1.0974754521	-0.0528938332
H	0.4568155790	-3.4456482801	0.1914748977
Ca	2.8600299749	-3.6228244506	-1.3743399196
O	1.4228776400	-4.2678537691	0.4086227038
H	0.9961173415	-5.1294734747	0.4592230211
O	3.4604909872	-1.6865066346	-0.4030280988
H	4.2609462943	-1.1864397992	-0.2354072808
H	2.3820070981	-1.2367929286	0.6357078764

TS24

21

Step = 0, E = 0.00000000

O	-0.8607433053	1.6492424812	-1.9826588999
O	1.0772239053	-0.3930112354	-2.5000691052
O	-1.3366627502	-1.3071181090	-0.1249530793
O	-2.8784917856	-1.9561255120	-2.2505148214
C	-0.4707339828	0.7098239805	-0.9888944721
C	0.9607315460	0.2336752055	-1.2324988674
C	-1.3857389265	-0.5032828034	-1.0390294830
C	-2.2801130029	-0.7157201789	-2.2317677776
H	-0.5417344781	1.1372578162	0.0157255279

H	1.6397533163	1.0876254360	-1.1501826946
H	1.2302887323	-0.5055632533	-0.4789757470
H	-1.6526605508	-0.5312818966	-3.1158697673
H	-2.9930520876	0.1302339424	-2.2234008382
H	-0.4457738792	2.4953583698	-1.7778871796
H	-3.7385295911	-2.0096458271	-1.3992538330
Ca	-2.4314596581	-3.5882549939	0.0090819120
O	-4.3874995385	-2.2293083550	-0.4173375476
H	-5.2595497639	-2.5327205058	-0.6819440861
O	-0.5979840670	-4.5174858286	-0.7249165451
H	0.1611875669	-4.1933494987	-1.2091764310
H	0.8065423009	0.2526507659	-3.1654762646

TS25

18

Step = 0, E = 0.00000000

O	-1.0301333714	1.7685125241	-2.0493602074
O	1.6339070025	-0.7573061309	-1.8466470956
O	-0.8724109816	-1.7796159962	-1.6295195842
O	-3.5909311802	-1.3933256141	-1.7262471511
C	-0.4007423581	0.5786227270	-1.6056573063
C	0.8741447163	0.3009238064	-2.4052896122
C	-1.3601820196	-0.5977667040	-1.7214093329
C	-2.7834051724	-0.4052586228	-1.7827088738
H	-0.1357441532	0.6487314782	-0.5438832623
H	0.5872096121	0.0837499026	-3.4425003437
H	1.4946049028	1.1985581252	-2.4044012328
H	-2.0242842633	-0.2873241594	-2.9338058257
H	-3.1394604572	0.6280650615	-1.7588242193
H	-0.5442992546	2.5177460449	-1.6859022644
Ca	-2.5541287289	-3.5555676659	-1.3600398075
O	-2.8315235453	-5.6073066021	-0.7797784119
H	-2.9046986228	-6.5118982617	-0.4860628599
H	0.9783763665	-1.4678636415	-1.7124334964

TS26

21

Step = 0, E = 0.00000000

O	0.7943557317	1.5785619064	-2.2377210583
O	2.1200826165	-1.7048857431	-1.7309880355
O	-0.7348167849	-1.6994801954	-2.8603714387
O	-1.9300616122	1.3744314917	-1.6592215615
C	0.6303150919	0.1774498086	-2.0145438253
C	1.9092437875	-0.5173437997	-2.4607241967

C	-0.6038796724	-0.3219147443	-2.7848676756
C	-1.8662928191	0.2968733054	-2.2119294453
H	0.4953679583	-0.0269805674	-0.9447579702
H	1.8587126145	-0.7192067044	-3.5371034728
H	2.7282803048	0.1952672215	-2.2954858527
H	-0.5101490750	0.1050306631	-3.7995232763
H	-2.7703593572	-0.3309093385	-2.3239060549
H	-0.0337272897	1.9912632879	-1.9506875689
Ca	0.7001378678	-4.0970667076	-2.2431221939
O	2.7638342064	-3.6747783514	-3.1254604282
H	3.6171128513	-4.0950561334	-2.9979560141
H	2.5802029082	-2.3978351939	-2.3283111007
O	-0.7783886670	-2.8954552122	-0.7838084079
H	-0.8450609106	-2.1766957079	-1.8042720654
H	-1.6569816554	-3.2376684484	-0.5949036796

TS27

21

Step = 0, E = 0.00000000

O	3.0109266372	1.2602884039	-0.4426703360
O	1.3616069530	-0.7530711324	-2.9772298861
O	2.8326458627	-2.3039784357	-1.2086916973
O	0.7390335025	2.5220141056	-1.7468176361
C	2.4069140989	0.1198595759	-0.9618270074
C	1.8034384838	0.4457442403	-2.3494029944
C	3.4270715597	-1.0200338929	-1.0830683556
C	0.6452134356	1.3936640051	-2.1793856250
H	1.5993364893	-0.2424748943	-0.3059413797
H	2.5850193819	0.9371929019	-2.9343245979
H	4.0156939092	-1.0237726978	-0.1653043018
H	4.1032273249	-0.8123602822	-1.9213205175
H	-0.3402875052	0.9763060468	-2.4550967052
H	1.4811486657	-0.6757839538	-3.9309429216
H	2.2453049159	-2.2447080249	-1.9750509826
Ca	2.2926228035	3.9185860020	-0.2214291504
O	2.6370966382	5.6280064090	-1.4765261264
H	3.0241479156	6.4782293755	-1.6760594435
O	1.7707827842	2.2392733698	1.3574669071
H	2.4629328005	1.5774306644	0.4856024692
H	2.1579559749	2.1579801399	2.2327654197

TS28

18

Step = 0, E = 0.00000000

O	2.6742124101	1.0327301599	0.2491654060
O	1.8026963107	-1.3805817514	-2.7048694191
O	2.9398808146	-2.4829986820	-0.4721102105
O	0.7581152976	1.9008600927	-1.6666787103
C	2.3759692894	-0.1354701582	-0.0757909162
C	1.9443580566	-0.0577115352	-2.2332074134
C	3.4589726847	-1.2005859825	-0.1835571942
C	0.7990313444	0.6916040080	-2.0228002342
H	1.3920791041	-0.5574183057	0.1859728277
H	2.9052034381	0.4099762352	-2.4186634874
H	3.9323999736	-1.2520757810	0.8022406261
H	4.2255397531	-0.8902334840	-0.9017873730
H	-0.1463647084	0.1239465977	-2.0844612845
H	1.9869571489	-1.4012409553	-3.6502872489
H	2.5477885771	-2.4131122877	-1.3596666113
Ca	1.9508166994	3.3174070710	-0.1442981804
O	2.3021947688	5.2952297645	0.6030799832
H	2.4258492277	6.1797809161	0.9377380146

REFERENCES

(1) Kästner, J. Umbrella Sampling. *WIREs Comput. Mol. Sci.* **2011,** *1*, 932-942.

(2) Barducci, A.; Bonomi, M.; Parrinello, M. Metadynamics. *WIREs Comput. Mol. Sci.* **2011,** *1*, 826-843.

(3) Maeda, S.; Harabuchi, Y.; Takagi, M.; Taketsugu, T.; Morokuma, K. Artificial Force Induced Reaction (AFIR) Method for Exploring Quantum Chemical Potential Energy Surfaces. *Chem. Rec.* **2016,** *16*, 2232-2248.

(4) Yang, L.; Liu, C.-W.; Shao, Q.; Zhang, J.; Gao, Y. Q. From Thermodynamics to Kinetics: Enhanced Sampling of Rare Events. *Acc. Chem. Res.* **2015,** *48*, 947-955.

(5) Horn, B. K. P. Closed-Form Solution of Absolute Orientation Using Unit Quaternions. *J. Opt. Soc. Am. A* **1987,** *4*, 629-642.
